# Is “sky” bluer than “grass” is green? Word–color associations dataset for cognitive science

**DOI:** 10.3758/s13428-026-03084-z

**Published:** 2026-06-17

**Authors:** Eldad Keha, Avishai Henik, Eyal Kalanthroff

**Affiliations:** 1 Department of Psychology, The Hebrew University of Jerusalem, Mt. Scopus, 91905 Jerusalem, Israel; 2 Department of Psychology, Achva Academic College, Arugot, Israel; 3 Department of Psychology and The Zelman Center for Brain Science, Ben-Gurion University of the Negev, Beer-Sheva, Israel; 4 Department of Psychiatry, Columbia University Medical Center, New York, NY USA

**Keywords:** Word–color associations, Color diagnosticity, Semantic Stroop effect, Memory colors, Dataset validation

## Abstract

Color plays a fundamental role in cognition, influencing perception, memory, and language processing. Although many studies have used word–color associations to examine cognitive processes, the selection of color-associated stimuli has often been arbitrary and rarely validated through independent behavioral measures. The current study developed and validated a dataset of color-associated object words for cognitive research. In Experiment 1, 298 participants provided subjective assessments for 143 words selected from 124 previous studies. For each word, participants indicated the most strongly associated color, rated the strength of the association, and selected the RGB value. The results revealed substantial variability across words in agreement and association strength. Based on these ratings, we constructed a dataset of 79 strongly color-associated words across 11 color categories. In Experiment 2, we validated the dataset in a UK sample (*N* = 568) using a manual semantic Stroop task, in which words were presented in congruent or incongruent colors. Dataset words produced a reliable semantic Stroop effect, with faster responses to congruent than incongruent color-associated word pairings, and this effect was larger for dataset than non-dataset words. In Experiment 3, we replicated this validation pattern in an independent US sample (*N* = 337), providing further evidence for the robustness and usefulness of the dataset for English-language cognitive research. Across the validation experiments, subjective association strength was related to the objective semantic Stroop effect. Together, the findings demonstrate that the current dataset provides an empirically validated set of color-associated words that can support research on perception, memory, semantic processing, and cognitive control.

**Supplementary Information:**

The online version contains supplementary material available at 10.3758/s13428-026-03084-z.

## Introduction

Objects in our environment are linked to specific colors. For example, we typically associate Grass with green and a Banana with yellow. Hering (1964) proposed that when we see familiar objects, we do not just see their colors as they are at that moment, but instead, we perceive them through the spectacles of *‘memory colors’*. These memory colors are formed through repeated presentation of the object in its color and are considered very stable. This means that our perception of an object’s color is influenced by our memory of its typical color from past experiences (Bartleson, 1960). This effect is most pronounced when the object is strongly associated with a particular color and when the observer is very familiar with it—a phenomenon known as “*color diagnosticity*” (Tanaka & Presnell, 1999). For instance, a ripe tomato might strongly evoke the color red because of the strong association between tomato and red and its high familiarity, making it highly color-diagnostic. Conversely, an object like a book is considered color-neutral since it comes in various colors. Empirical research that supported this idea has consistently shown that objects presented in their typical colors are recognized more quickly and accurately than when they appear in atypical colors (e.g., Tanaka & Presnell, 1999; Naor-Raz et al., 2003) or black and white (Rossion & Pourtois, 2004). This advantage was most pronounced for highly color-diagnostic objects—such as a yellow banana—where color serves as a crucial identifying feature. Similarly, Connell and Lynott (2009) demonstrated that naming the ink color of written words was faster not only when the meaning of the word was associated with the ink color (e.g., the word BEAR in brown ink) but also when the context is associated with the presented ink color (e.g., BEAR in white ink, following the sentence “Joe was excited to see a bear at the North Pole”). These findings highlight the role of color in object recognition.

Color-associated words are widely used as cues in cognitive research to study attention, memory, visual perception, and semantic processing (Augustinova et al., 2019; Burt, 2002; Kinoshita et al., 2018; Mani et al., 2013; Naor-Raz et al., 2003). Perhaps the most widely used application of color-related words in cognitive science is in the color-word Stroop task (Stroop, 1935). In this task, participants are asked to identify the ink color of words that could be either congruent (e.g., RED written in red), incongruent (e.g., BLUE written in red), or neutral (e.g., BOOK written in red). In addition to measuring the interference effect (slower reaction times (RTs) for incongruent compared to neutral trials) and the facilitation effect (faster RTs to congruent trials than neutral trials), researchers have also measured the *semantic* *Stroop* *effect*, which reflects delayed responses to color-associated words (e.g., SKY, GRASS, TOMATO) when they appear in atypical colors (SKY written in red) versus when they appear in their typical colors (SKY written in blue) or when non-color-related words appear (BOOK written in blue; Augustinova et al., 2019; Klein, 1964). The semantic Stroop effect^1^ indicates that automatic semantic processing is triggered by the meaning of the color-related written word, and not only supports the color diagnosticity effect but also provides a way of measuring it.

One defining characteristic of research using color-associated words is the variability in stimulus selection, as researchers often use different, arbitrary sets of stimuli. Many studies using color-associated words rely on the assumption that these words reliably activate the intended color representations. However, if the strength and consistency of these associations vary substantially across words, then differences between studies may reflect differences in stimulus validity rather than differences in the underlying cognitive mechanisms being tested. This is important for theories of semantic activation, color knowledge, memory colors, object recognition, and cognitive control, all of which depend on the assumption that color-associated words automatically activate color-related representations. Hence, developing a dataset for highly color-diagnostic words is crucial for ensuring reliability, consistency, and accuracy across experiments. Most previous attempts to establish datasets of color-associated words have focused on large sets of words that are not necessarily linked to objects with intrinsic colors (Chen et al., 2020; Ikoma et al., 2020; Jahanian et al., 2017), thereby limiting their applicability for studying memory colors and color-object associations. Prior efforts to build datasets for high color-diagnostic objects, such as those by Scheibe et al. (1967) and Solso (1971), are outdated, rely on small sample sizes, and do not employ objective measures to quantify word–color associations. To provide a precise and ecologically valid dataset, the current study focuses exclusively on object words with strong color associations that were used in 124 previous studies. In Experiment 1, we assessed agreement levels, association strength, and exact red, green, and blue (RGB) values for 143 color-diagnostic words and established criteria for inclusion in the dataset. Next, in Experiment 2, we tested the correlation between subjective reports and the semantic Stroop effect and compared this effect between color-associated words that showed a strong association in Experiment 1 and those that did not.

## Experiment 1 – Developing a color-related words dataset

### Method

#### Participants

Three hundred participants took part in the experiment in return for a small monetary compensation (2.7 GBP). All participants had normal or corrected-to-normal vision, were not color-blind, were native English speakers, and were born and currently living in the UK. Two participants were excluded from the analysis for random responding, reflected in very fast responses that were inconsistent with the general pattern of agreement, and therefore, the analyzed sample comprised 298 participants, 180 females, 114 males, two individuals identifying as another gender (non-binary, agender, etc.), and two who preferred not to report gender. Because Experiment 1’s goal was to assemble a color-word dataset rather than to test a specific hypothesis, no statistical power calculation was conducted for Experiment 1.

#### Materials and stimuli

One hundred forty-three color-associated words (see Supplementary A) were selected from 124 previous studies that used color-words (the full list of studies, as well as the study-inclusion process, is provided in Supplementary B). The initial pool of color-associated words was constructed through a structured literature-based search. We first searched for cognitive-science studies that used color-associated object words, using the following search terms: “*semantic Stroop*,” “*color-associated words*,” “*color diagnosticity*,” “*memory color*,” “*object-color associations*,” and “*color-word associations*.” We then used citation chaining by examining both the reference lists of the identified papers and papers that cited them. This process was conducted in two rounds to identify additional relevant studies. All candidate papers were then manually screened, and words were extracted only from studies that used concrete color-associated object/concept words as experimental stimuli. Studies focusing primarily on abstract color meanings, affective color associations, or stimuli outside the scope of cognitive color-word research were excluded. This process resulted in 143 words, which were sufficient for the initial pool.

**Fig. 1:**
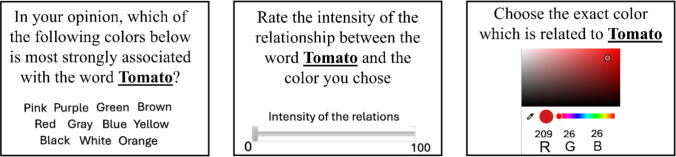
Example trial from Experiment 1. *Note.* A schematic representation of the three questions of Experiment 1. A color picker was used to report the exact color that is associated with the word

Participants were asked to respond to three questions for 76 randomly selected color-associated words from the list (see Fig. 1):

1. “In your opinion, which of the following colors below is most strongly associated with the word ______?“ (pick one out of 11 possible colors; red, blue, green, yellow, pink, black, white, grey, purple, orange, brown), which aligned with the 11 basic color terms identified by Berlin and Kay (1991). The order of the color options was fixed across trials and participants.
2. “Rate the intensity of the relationship between the word ______ and the color you chose” (position the marker along a 0–100 scale, initially centered).
3. Using the computer mouse, participants used a color picker, encompassing all possible RGB combinations, to select the exact color they associated with the word.

#### Procedure

The study was approved by the university’s ethical committee (HUJI-2023-06011). Participants received a link to the questionnaire through a Prolific online research platform. After signing an informed consent form, participants were asked to complete a demographic questionnaire, which consisted of questions regarding their age, years of education, gender, and native language. Next, each participant completed a random selection of 76 trials (out of 143 possible trials),^2^ which included a target word (presented in black ink), and the three questions mentioned above (Fig. 1). At the end of the experiment, participants were thanked and debriefed. The average time to complete the study was 29 min and 13 s.

### Results

We first calculated the mean percentage of associated color agreement (Question 1) for each word in the set. While the mean percentage of agreement was high at 80.64, there was considerable variance across words, ranging from 36.11% to 100%, with a standard deviation (SD) of 17.48. Mean values and SDs of color association strength (Question 2) were calculated for each word. Consistent with the mean percentage of agreement, the average association strength was high at 78.88, but there was again considerable variance between words, ranging from 57.16 to 98.19, with a standard deviation of 9.05. We examined the relation between the percentage of agreement and association strength across words. These two subjective measures were strongly correlated, *r*(134) =.645, *p* <.001, 95% CI [.535,.734], indicating substantial overlap between them. Finally, the mean RGB values (Question 3) were also calculated for each word, based on the exact color that was chosen on the color picker. The word-by-word results for all three questions are presented in Supplementary A (for a similar detailed table of the second and third most associated colors for each word, see Supplementary C)^3^.

To establish the dataset, we applied the following thresholds: an association was deemed strong if it fell within the upper-quartile for both agreement level (≥75%) and association strength (mean ≥75). These criteria balanced the need for strong associations while ensuring a comprehensive dataset. The final list of words that met these thresholds and were included in the dataset is presented in Table 1.

**Table 1:** The color-related words dataset

Color	Color-related words
Black	Coal, Crow, Night
Blue	Smurf, Ocean, Sky, Sea, Jeans, Pool
Brown	Mud, Chocolate, Coffee, Cocoa, Cigar, Dirt, Bear
Green	Grass, Spinach, Pea, Broccoli, Lime, Lettuce, Cucumber, Avocado, Leaf, Cabbage, Zucchini, Cactus, Bush, Crocodile, Kiwi, Field, Frog
Grey	Elephant, Concrete, Rhinoceros, Hippopotamus, Shark, Seal, Smoke
Orange	Orange, Carrot, Pumpkin, Goldfish
Pink	Flamingo, Salmon, Pig, Shrimp, Tongue, Barbie
Purple	Eggplant, Plum
Red	Blood, Tomato, Strawberry, Ketchup, Fire Engine, Stop Sign, Cherry, Poppy, Heart
White	Snow, Swan, Bride, Chalk, Clouds, Bone, Cauliflower
Yellow	Lemon, Banana, Sun, Yolk, Mustard, Corn, Chick, Butter, Canary, Cheese, Pineapple

The color-related words dataset includes color-related words with a percentage of agreement of at least 75% and a mean association strength of at least 75. Each word is associated with the most commonly reported color (Question 1), which consistently corresponds with the mean RGB values (Question 3)

## Experiment 2 – Validating the color-related words dataset in a UK sample

In Experiment 1, we developed a word–color association dataset by extracting both the mean association strength and the corresponding mean RGB values for each word. In Experiment 2, we validated this dataset using the semantic Stroop effect—words were presented either in their congruent color (mean RGB value), or an incongruent color and RT differences between these conditions were calculated (Kinoshita et al., 2018; Klein, 1964). This effect reflects the semantic association between words and their respective colors (Neely & Kahan, 2001) and allowed us to evaluate the validity of the color-word dataset. For that aim, we used two complementary methods: first, we assessed the correlation between subjective association strengths from Experiment 1 and the semantic Stroop effect. Second, we compared the semantic Stroop effect for words that met the dataset inclusion threshold in Experiment 1 with those that did not.

### Method

#### Participants

Five hundred eighty-three participants, who did not take part in Experiment 1, took part in this experiment in return for a small monetary payment (4 GBP). Inclusion/exclusion criteria, recruitment procedures, and data-filtering thresholds were identical to Experiment 1. Specifically, nine participants were excluded for falling more than 3 SDs below the sample’s mean accuracy rate, and six participants were excluded for falling more than 3 SDs above the sample’s mean RT. Therefore, the final sample included 568 participants. The participants had a mean age of 41.65 years (SD = 14.86). The sample comprised 284 females, 243 males, six individuals identifying as another gender (non-binary, agender, etc.), and 35 who preferred not to report gender. Because Experiment 2’s goal was to validate a color-word dataset rather than to test a specific hypothesis, no statistical power calculation was conducted for Experiment 2. Instead, the relatively large sample was chosen to obtain stable item-level estimates of the semantic Stroop effect across a broad set of candidate words. Such stable estimates were important for later validating the dataset, examining word-level behavioral effects, and comparing dataset and non-dataset items.

#### Materials and Stimuli

##### Stimuli

In Experiment 2, we utilized all the color-related words from Experiment 1, with the exclusion of seven words associated with the color black, as black was used as the background color in Experiment 2 (thus, 136 color-associated words were included; see Supplementary E sheets). Ten color categories were included: red, blue, green, yellow, pink, white, grey, purple, orange, and brown. Each word was presented in its congruent color, based on its mean RGB value obtained from responses to Question 3 in Experiment 1 (which was always consistent with its identified color category), or in incongruent colors, which were the mean RGB of different stimuli, not from the same color category. The experiment was designed using PsychoPy (v2024.1.5) and deployed online via the Pavlovia platform. The stimuli were presented at the center of the screen, in Open Sans font at a height of 0.1 PsychoPy units, against a black background. Timing precision and randomization were controlled through custom Python scripts within PsychoPy. Participants completed the task on their personal computers, with the experiment running in full-screen mode to minimize distractions and ensure standardized viewing conditions.

##### List construction

Ten lists were randomly constructed, each including 16 color-related words from four different color categories (four words for each color category; the ten lists can be found in Supplementary E sheets). Each word was presented in either its congruent color or in an incongruent color, which was a different color from the same list. For example, if the words *Banana* and *Grass* were randomized on the same list, *Banana* could appear either in its own congruent RGB value (e.g., 241, 231, 35, reflecting yellow) or in *Grass*’s congruent color (e.g., 54, 168, 37, reflecting green) and vice versa. Eventually, each list comprised 32 stimuli—16 congruent and 16 incongruent—presented 8 times throughout the experiment (256 trials in total). Because the number of available words was not identical across color categories, a few stimuli had to be included in two, rather than just one, lists to construct balanced lists with four response colors and an equal number of words per color. The complete stimulus lists, including the repeated items, color assignments, congruent and incongruent pairings, and full list structure, are provided in Supplementary E and available at the following OSF link: https://osf.io/twqys/?view_only=5a1b08637fd743838dfa5ce8070f0e29.

#### Procedure

Participants were recruited via Prolific and completed a semantic Stroop task. The procedure began with a practice phase in which participants identified the color of an asterisk to learn which key corresponded to each color. Responses were made manually using the top-row number keys 1, 2, 3, and 4. Participants were instructed to place four fingers of their dominant hand on these keys throughout the task. The practice phase included 40 trials that were identical in structure to the experimental trials and was designed to train participants to associate each response key with the correct color. Each participant was randomly assigned to one of ten possible sets of stimuli (for full details on the construction of the list, see Supplementary E). Following the practice phase, participants completed 256 experimental trials. Each trial consisted of a 500-ms white fixation cross, followed by a 250-ms black screen interval, after which the stimulus appeared for 2750 ms or until a response was made.

### Results

**Fig. 2:**
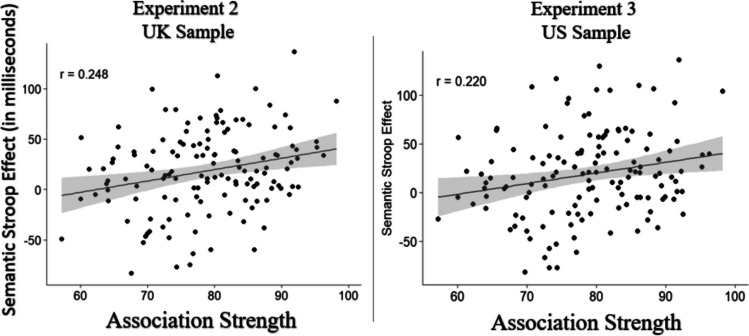
Correlation between subjective association strength and the semantic Stroop effect. *Note.* The *left panel* presents the correlation between the subjective measure of association strength (Question 2; Experiment 1) and the objective measure of the semantic Stroop effect in the UK sample (Experiment 2). The *right panel* presents the same correlation in the US sample (Experiment 3)

Accuracy levels were very high (M = 96.6, SD = 2.6), and no modulation by condition was found. Therefore, we focused our analysis on RT data. First, we tested whether the semantic Stroop effect correlated with the subjective reports of Experiment 1, and specifically, with the percentage of agreement (Question 1) and association strength (Question 2) from Experiment 1. The semantic Stroop effect was calculated for correct trials of each word separately as incongruent RT – congruent RT. Trials with RTs exceeding 3.5 median absolute deviations from the median of each participant in each congruency condition were excluded (see Table 2 for exclusion summary by condition). The Pearson correlation analyses yielded significant correlations between the semantic Stroop effect and both the percentage of agreement, *r*(134) =.180, *p* =.036, and association strength, *r*(134) =.248, *p* =.003 (see Fig. 2).

**Table 2:** Descriptive statistics for RTs and accuracy by experiment, word-type, and congruency

Word-type	Congruency	RT, M (SD)	ACC, M (SD)	RT-trimmed trials (%)
Experiment 2				
Dataset	Congruent	731 (31.4)	96.4 (1.34)	4.1
Dataset	Incongruent	758 (31.8)	95.9 (1.85)	3.9
Non-dataset	Congruent	741 (29.5)	96.1 (1.38)	4.6
Non-dataset	Incongruent	748 (33.3)	95.7 (1.43)	3.8
Experiment 3				
Dataset	Congruent	753 (32.5)	95.8 (1.69)	4.4
Dataset	Incongruent	782 (34.0)	95.3 (1.88)	4.3
Non-dataset	Congruent	772 (34.8)	95.7 (1.68)	5.1
Non-dataset	Incongruent	780 (38.2)	95.5 (1.93)	4.4

RT = reaction time in milliseconds; ACC = accuracy percentage. Values represent word-level means, with standard deviations in parentheses. Dataset words refer to words that met the inclusion criteria in Experiment 1; non-dataset words refer to words that were tested but did not meet these criteria. RT-trimmed trials indicate the percentage of trials excluded by the RT-trimming procedure within each word-type × congruency condition.

**Fig. 3:**
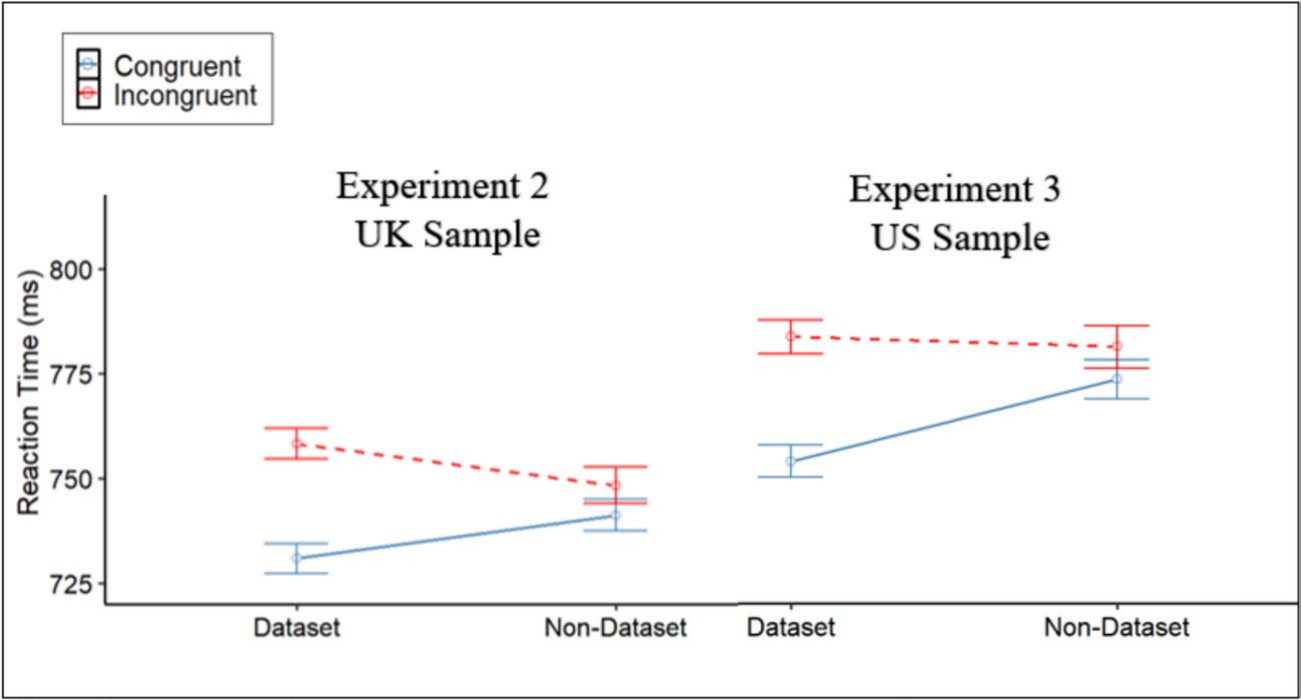
The semantic Stroop effect for dataset and non-dataset words. *Note.* Mean reaction times (in milliseconds) as a function of congruency (congruent vs. incongruent) and word-type (dataset vs. non-dataset) in Experiments 2 (UK sample) and 3 (US sample). *Error bars* indicate ±1 standard error of the mean

Next, we compared the semantic Stroop effect for words that met the inclusion threshold in Experiment 1 with those that did not. A two-way mixed ANOVA was conducted at the item-level, with congruency condition (congruent vs. incongruent) as a within-item variable and word-type (dataset vs. non-dataset) as a between-item variable. Words were classified as dataset or non-dataset items based on the inclusion criteria from Experiment 1. Each participant was randomly assigned to one of the stimulus lists, and each list included words that varied in whether they met these criteria. Therefore, the comparison between dataset and non-dataset words was conducted at the item-level, with word-type treated as a between-item factor. The results yielded a main effect for congruency condition, *F*(1, 134) = 25.795, *p* <.001, ${\upeta}_{p}^{2}$=.161, indicating longer RTs for incongruent compared to congruent trials (the semantic Stroop effect). There was no significant difference in RTs between the dataset and non-dataset word-types, *F*(1, 134) = 0.001, *p* =.970. However, a significant congruency × word-type interaction was observed, *F*(1, 134) = 8.966, *p* =.003, ${\upeta}_{p}^{2}$=.063 (see Fig. 3). This interaction indicated that the semantic Stroop effect was stronger for words included in the dataset (*M *= 27 ms; *SD* = 38 ms) than for words not included in the dataset (*M* = 7 ms; *SD* = 41 ms). Planned comparisons revealed that the semantic Stroop effect was significant for words included in the dataset, *t*(134) = 6.077, *p* <.001, Cohen’s *d*_*z*_* =*.525, but not significant for words that did not meet the dataset criteria, *t*(134) = 1.394, *p* =.165 (see Table 2).

## Experiment 3 – Replication and generalizability in a US sample

Experiment 3 was conducted to further validate the color-related words dataset and to examine whether the findings of Experiment 2 generalize beyond the original UK sample. This issue is important because color-object associations may vary across cultures and populations, and a useful stimulus dataset should produce reliable effects across independent samples. Therefore, in Experiment 3, we tested a new sample of English-speaking participants who were born and raised in the United States using the same semantic Stroop validation procedure as in Experiment 2.

### Method

#### Participants

Three hundred forty-nine participants, who did not take part in Experiment 1 or 2, took part in this experiment in return for a small monetary payment (4 GBP). Inclusion/exclusion criteria, recruitment procedures, and data filtering thresholds were identical to Experiment 1 and Experiment 2. Five participants were excluded for falling > 3 SDs below sample’s mean accuracy rate, and seven participants were excluded for falling > 3 SDs above sample’s mean RT. Therefore, the overall sample included 337 participants. The participants had a mean age of 41.24 years (SD = 13.39). The sample comprised 198 females, 134 males, four individuals identifying as another gender (non-binary, agender), and 1 who preferred not to report gender.

#### Materials, stimuli, and procedure

The materials, stimuli, experimental procedure, and data-processing criteria were identical to those used in Experiment 2.

### Results

The analysis model, RT-trimming procedure (see Table 2 for exclusion summary by condition), and exclusion criteria were identical to those used in Experiment 2. Accuracy levels were very high (*M* = 95.5, *SD* = 3.86), with no modulation by condition; therefore, RTs were the primary dependent measure. The Pearson correlation analyses yielded a marginal correlation between the semantic Stroop effect and percentage of agreement, *r*(134) =.166, *p* =.054, and a significant correlation between the semantic Stroop effect and association strength, *r*(134) =.218, *p* =.011.

Next, we compared the semantic Stroop effect for words that met the inclusion threshold in Experiment 1 with those that did not. A two-way mixed ANOVA was conducted with congruency condition (congruent vs. incongruent) as a within-item variable and word-type (dataset vs. non-dataset) as a between-item variable. The results yielded a main effect for congruency condition, *F*(1, 134) = 23.383, *p* <.001, ${\upeta}_{p}^{2}$=.149, indicating longer RTs for incongruent compared to congruent trials (the semantic Stroop effect). There was no significant difference in RTs between the dataset and non-dataset word-types, *F*(1, 134) = 3.263, *p* =.073, ${\upeta}_{p}^{2}$=.024. However, a significant congruency × word-type interaction was observed, *F*(1, 134) = 8.072, *p* =.005, ${\upeta}_{p}^{2}$ =.057. This interaction indicated that the semantic Stroop effect was stronger for words included in the dataset (*M* = 29 ms, *SD* = 41 ms) than for words not included in the dataset (*M* = 8 ms, *SD* = 46 ms). Planned comparisons revealed that the semantic Stroop effect was significant for words included in the dataset, *t*(134) = 5.779, *p* <.001, Cohen’s d_z_ = 0.500, but not significant for words that did not meet the dataset criteria, *t*(134) = 1.334, *p* =.185 (see Table 2).

### Comparison with previous semantic Stroop effects

To contextualize the magnitude of the semantic Stroop effects observed in the current study, we compared the effects obtained for the validated dataset words in Experiments 2 and 3 with effects reported in previous studies using manual semantic Stroop or Stroop-like color-association paradigms. Out of the 124 studies identified for our initial word pool (see method section of Experiment 1), we focused on studies that used manual responses, as in the present investigation, because response modality is known to influence the magnitude of semantic Stroop effects (Augustinova et al., 2019; Kinoshita et al., 2018). In Table 3, we included studies that measured either the semantic Stroop effect, defined as the difference between color-associated words presented in incongruent versus congruent colors, or the semantic interference effect, defined as the difference between color-associated words presented in incongruent colors and neutral words presented in the same colors. This resulted in 13 effect estimates from nine previous studies.

**Table 3:** Comparison of standardized semantic Stroop and semantic interference effects across manual response studies

Source	Experiment/Group	Tested effect	Cohen’s *d*_av_	Cohen’s *d*_z_
**Current study**	Experiment 2, Dataset words	Semantic Stroop effect	0.22	0.55
**Current study**	Experiment 2, Non-dataset words	Semantic Stroop effect	0.10	0.19
**Current study**	Experiment 3, Dataset words	Semantic Stroop effect	0.23	0.59
**Current study**	Experiment 3, Non-dataset words	Semantic Stroop effect	0.12	0.22
Schmidt & Cheesman (2005)	Experiment not specified	Semantic Stroop effect	NA	0.40
White et al. (2016)	Experiments 1/2	Semantic interference effect	NA	0.16
White et al. (2016)	Experiments 3/4	Semantic interference effect	NA	0.19
Augustinova et al. (2018)	Younger-adult group	Semantic interference effect	0.13	NA
Kinoshita et al. (2018)	Experiment 2, Low-control condition	Semantic interference effect	0.14	NA
Kinoshita et al. (2018)	Experiment 2, High-control condition	Semantic interference effect	0.08	NA
Augustinova et al. (2019)	Experiment 1	Semantic interference effect	0.22	NA
Augustinova et al. (2019)	Experiment 2	Semantic interference effect	0.19	NA
Scaltritti et al. (2022)	Experiment not specified	Semantic interference effect	0.10	0.32
Sulpizio et al. (2022)	Experiment not specified	Semantic interference effect	NA	0.48
Sulpizio et al. (2024)	Experiment 1A	Semantic interference effect	0.05	NA
Sulpizio et al. (2024)	Experiment 2A	Semantic interference effect	0.13	0.33
Qiu and van Heuven (2025)		Semantic Stroop effect	0.33	0.81

Studies from the current manuscript are presented first followed by previous manual response studies in chronological order. Dataset words refer to words that met the inclusion criteria in Experiment 1. Non-dataset words refer to words that were tested but did not meet these criteria. The semantic Stroop effect refers to the contrast between color-associated words presented in incongruent versus congruent colors. The semantic interference effect refers to the contrast between color-associated words presented in incongruent colors and color-neutral words presented in the same colors. Cohen’s *d*_z_ was calculated from the relevant *t* value when available. Cohen’s *d*_av_ was calculated from condition means and standard deviations when available, following Lakens (2013). NA indicates that the necessary data were not available for that effect-size estimate

For each study, we extracted or calculated two standardized effect-size estimates when the necessary information was available: Cohen’s *d*z and Cohen’s *d*av.^4^ Because previous studies reported different statistics, effect sizes were derived from the information available in each article. When *t* values were available for the relevant within-participant or within-item comparison, Cohen’s *d*_z_ was calculated as *t*/√*n*, where *n* refers to the number of participants or items entering the contrast. When condition means, and standard deviations were available, Cohen’s *d*_av_ was calculated as the mean difference between conditions divided by the average of the two condition-specific standard deviations. We report both indices when possible because *d*_*z*_ and *d*_av_ use different standardizers: *d*_z_ standardizes the effect by the variability of the paired differences, whereas *d*_av_ standardizes the effect by the average variability of the two repeated-measures conditions. This distinction is important for interpreting and comparing repeated-measures effects across studies (Lakens, 2013). For the current study, effect sizes were calculated from the raw participant-level data in each validation experiment to make them comparable to the indices extracted from previous studies. In Experiments 2 and 3, the semantic Stroop effect was defined as the difference between incongruent and congruent color-associated word pairings. This effect was calculated separately for dataset and non-dataset words. For each experiment and word-type condition, we calculated Cohen’s *d*z by dividing the mean paired difference by the standard deviation of the paired differences, and Cohen’s *d*av by dividing the same mean difference by the average of the standard deviations of the congruent and incongruent conditions. These calculations enabled the effects observed in the current study to be reported using the same repeated-measures effect-size indices extracted or reconstructed from previous manual response studies. Because the goal of Table 3 was to compare the current effects with previous participant-level studies, the current-study effect sizes in Table 3 were calculated from participant-level data. Therefore, these values differ slightly from the item-level planned-comparison effect sizes reported in the Results sections. As shown in Table 3, the semantic Stroop effects observed for the dataset words in both Experiments 2 and 3 were larger than those typically reported in previous manual response studies.

## General discussion

In this study, we aimed to develop a reliable dataset of color-related words for research on color perception. In Experiment 1, we constructed the dataset by administering a subjective questionnaire to a large group of participants. While the list of words for this study was drawn from previous studies, our results showed a high variability—some words were clearly and strongly associated with a single color, while others showed weaker associations. Using a threshold based on the upper quartile for both agreement level and association strength, we have developed the final dataset (Table 1). In Experiments 2 and 3, we validated these associations using the semantic Stroop effect as an objective RT-based measure. Our findings confirmed the dataset’s validity, first by showing a significant correlation between the semantic Stroop effect and the subjective reports in Experiment 1, and second by showing that the semantic Stroop effect was significantly stronger for words that were included in the dataset compared to those that were not included.

Our results revealed substantial variability in how different words are associated with colors, both across different words and among individuals, emphasizing the complexity and individuality of color-object associations. This variability underscores the importance of our findings, particularly in light of the current state of research. In existing literature, across various fields—such as the color-word Stroop task, object recognition, and color constancy—the selection of color-related object stimuli has often been arbitrary, based largely on the individual judgment of the researcher. As suggested by our results, these arbitrary selections may contribute to differences between studies. Our study addresses this gap by providing a dataset of stimuli, offering a more reliable foundation for research in these areas. By building this dataset, we not only enhance the reliability of future studies but also contribute to the standardization of experimental materials, allowing for more meaningful comparisons across studies. Because theoretical conclusions in studies using color-associated words depend on the assumption that these words reliably activate color representations, validating these associations improves the construct validity of research on semantic activation, color knowledge, object recognition, and cognitive control.

In the present study, we specifically focused on color-related object words with high diagnosticity, meaning that these words exhibited strong and consistent associations with particular colors. This high agreement on color associations allowed us to construct a tailored dataset for investigating cognitive processes related to color semantics. In contrast, previous research has aimed to establish mappings for words with low color diagnosticity, often employing large-scale datasets and computational models to quantify the degree of association between words and colors (e.g., Chen et al., 2020; Jahanian et al., 2017). For example, words such as “justice” or “honesty” do not inherently evoke a strong association with a specific color, but efforts have been made to computationally estimate and map their color relationships using probabilistic models and topic-based approaches (Jahanian et al., 2017). Furthermore, studies on color–word embeddings have demonstrated that certain warm colors (e.g., red, orange) and cool colors (e.g., blue, green) carry semantic connotations that extend beyond perceptual features, influencing language processing and conceptual organization (Ikoma et al., 2020). The approach used in the current study differs from prior studies by focusing on high-color-diagnosticity words, ensuring that our dataset is optimized for studying color associations in a more controlled, semantically precise manner. This distinction is crucial, as research using general word–color mappings often includes a broad range of concepts with variable levels of color association, whereas our dataset is specifically structured to explore color-related semantic processes for words that represent objects/concepts that naturally appear in certain colors and strongly evoke specific colors.

Experiment 3 also addresses an important question about the dataset’s generalizability to an English-speaking population. The original dataset was constructed using participants from the UK, and color-object associations may vary across cultures and languages (Pettersson, 1982; Turganbayeva et al., 2014). However, the replication of the validation effects in a new sample of participants from the United States suggests that the dataset is not limited to the original UK sample. Importantly, the effect sizes of the semantic Stroop effect for dataset words were highly similar across Experiments 2 and 3 (see Table 3), and the correlations between subjective association strength and the semantic Stroop effect were of comparable magnitude across the two samples. These findings support the generalizability of the dataset as an English-language resource for cognitive research. At the same time, this evidence should not be taken to imply full cross-cultural or cross-linguistic generalizability. Future research should therefore extend and validate the dataset in additional languages, cultures, and populations to determine which color-object associations are universal and which are shaped by cultural or linguistic experience. The current study not only offers a validated dataset to test this question but also provides a method that can be used to generate and validate such databases in other languages and cultures.

The semantic Stroop effects observed for the validated dataset words were also notable when compared with previous manual response studies. As shown in Table 3, the effects obtained for dataset words in both validation experiments were larger than the effects reported in most previous studies using manual semantic Stroop or semantic interference contrasts. This comparison should be interpreted cautiously because the studies differed in their exact contrasts, stimulus sets, response mappings, and task structures. Nevertheless, it provides an important benchmark. Previous work has shown that semantic effects in manual color-identification tasks are often smaller and more variable than in vocal-response tasks (Augustinova et al., 2019; Sharma & McKenna, 1998; Keha & Kalanthroff, 2023), and in some cases become reliable mainly when control is reduced, for example by increasing the frequency of non-word neutral trials (Kinoshita et al., 2018). At the same time, semantic interference has also been observed in manual response tasks without such manipulations (e.g., Scaltritti et al., 2022; Sulpizio et al., 2022). However, as shown in Table 3, these effects were generally small compared with the effects observed for the validated dataset words in Experiments 2 and 3. Thus, the present results suggest that empirically selecting words with strong and reliable color associations can increase the magnitude and robustness of these effects.

The dataset developed in the current study comes with certain limitations. One limitation of the present validation approach is that the semantic Stroop effect was defined as the difference between congruent and incongruent color-associated word pairings. This contrast is useful for validating the dataset because both facilitation from typical color pairings and interference from atypical color pairings are informative about the strength of the underlying object-color association. However, this contrast does not allow us to determine the separate contributions of semantic facilitation and semantic interference. Future studies using the present dataset could include color-neutral words, allowing researchers to decompose the overall semantic Stroop effect into facilitation and interference components. Second, in Experiments 2 and 3, the incongruent condition was limited to a subset of color-associated word pairings, which, despite careful selection, may have varied in psychological distance, potentially influencing the magnitude of the semantic Stroop effect. In addition, words associated with the color black were not assessed in Experiments 2 and 3, given that the task’s background color was black. To conclude, the current study provides a carefully constructed and validated dataset of high-color-diagnosticity words that can be used in various cognitive studies. By demonstrating a strong link between subjective color associations and the objective semantic Stroop effect, our findings highlight the robustness of these associations in shaping semantic processing. Finally, the current study provides a reliable tool for investigating color-related cognitive processes and for future research that aims to expand this stimulus dataset to other languages or incorporate different types of stimuli, such as images.

## Supplementary Information

Below is the link to the electronic supplementary material.

Supplementary file1 (DOCX 2903 KB)

## Data Availability

The datasets generated and/or analyzed during the current study are available in the OSF repository, https://osf.io/twqys/?view_only=5a1b08637fd743838dfa5ce8070f0e29.

## Footnotes

1 In the present study, we use the term semantic Stroop effect to refer to the congruency contrast between color-associated words presented in their typical color and the same type of words presented in an atypical color. This contrast differs from semantic interference, which is more narrowly defined as the difference between color-associated words presented in an atypical color and color-neutral words presented in the same color. Although semantic interference provides a more direct estimate of interference, the congruency contrast was most suitable for the present validation purpose because both faster responses to typical color-associated word pairings and slower responses to atypical pairings reflect the strength of object–color associations.

2 Note that due to the length of the experiment, each participant was randomly presented with half of the possible color-related words.

3 See Supplementary D for a detailed graph that describes the mean association strength for each of the 11 color categories.

4 We report both Cohen’s *d*_z_ and Cohen’s *d*av because not all previous studies reported the same information. Cohen’s *d*_z_ can be calculated directly from the *t* value and sample size for a paired comparison, but some studies did not report the relevant *t* test and instead provided only condition means and standard deviations. In those cases, Cohen’s *d*av could be estimated from the reported descriptive statistics by dividing the mean difference between conditions by the average of the two condition-specific standard deviations. Thus, reporting both indices allowed us to include a broader set of studies while maintaining transparency about how each effect-size estimate was derived.

## Code availability

All analysis and stimulus-generation code are available at https://osf.io/twqys/?view_only=5a1b08637fd743838dfa5ce8070f0e29.
