# Supplementary material for: Is “sky” bluer than “grass” is green? Word–color associations dataset for cognitive science
Source: Behav Res Methods. 2026 Jun 17;58(7):202. doi: 10.3758/s13428-026-03084-z (PMC13275740; doi:10.3758/s13428-026-03084-z)
Supplement: Supplementary file 1 — Supplementary file1 (DOCX 2903 KB) [file 13428_2026_3084_MOESM1_ESM.docx]

**Supplementary Materials**

***Supplementary Materials Overview***
 The Supplementary Materials provide detailed item-level and summary-level information for the color-related word dataset. These materials are intended to support researchers who wish to select stimuli for future studies involving color associations, semantic Stroop effects, object-color knowledge, or related cognitive processes. To improve usability, the supplementary sections are organized from the most central information to more detailed information. Supplementary A provides the primary item-level information for each word, including its most strongly associated color. Supplementary B provides a list of previous studies that used color-associated stimuli or related semantic Stroop materials. Supplementary C provides additional information about secondary and tertiary color associations, which may be useful for researchers who wish to avoid unintended color associations or select alternative color pairings. Supplementary D provides a summary of the association strength across color categories. Supplementary E provides the full list structure used in Experiments 2 and 3. Together, these materials allow readers to evaluate both the item-specific properties of individual words and the broader structure of color associations across the dataset.

**Supplementary A** Supplementary A presents the main item-level information for all color-related words included in the study. To improve readability and make the dataset easier to navigate, the information is divided into two tables. Supplementary A1 presents the words included in the final dataset, whereas Supplementary A2 presents the words that were included in the broader stimulus pool but did not meet the dataset inclusion criteria. For each word, the tables report the color that was most strongly associated with that word, the percentage of participants who selected or reported that color, the mean association strength, the standard deviation of the association-strength ratings, and the corresponding RGB values. The words in each table are numbered independently so that dataset and non-dataset items can be inspected separately. Together, these tables serve as the primary reference for researchers who wish to select color-associated words for experimental use, while also retaining transparency regarding the broader item pool and allowing researchers to consider alternative stimuli.

**Supplementary A1**

***Dataset Words***

| **No.** | **Word** | **Associated Color** | **% Of Agreement** | **Mean Association Strength (SD)** | **RGB Values** |
| --- | --- | --- | --- | --- | --- |
| 1 | Avocado | Green | 91.39 | 82.9 (19.37) | 79, 148, 53 |
| 2 | Banana | Yellow | 99.32 | 92.53 (14.14) | 241, 231, 35 |
| 3 | Barbie | Pink | 94.74 | 77.39 (23.74) | 235, 98, 188 |
| 4 | Bear | Brown | 85.71 | 76.93 (21.35) | 106, 60, 25 |
| 5 | Blood | Red | 98.67 | 95.27 (9.23) | 212, 18, 16 |
| 6 | Bone | White | 86.75 | 80.42 (21.76) | 241, 238, 232 |
| 7 | Bride | White | 97.26 | 87.27 (17.03) | 249, 248, 247 |
| 8 | Bush | Green | 95.24 | 79.09 (18.01) | 53, 125, 36 |
| 9 | Butter | Yellow | 97.28 | 83.23 (19.68) | 238, 233, 90 |
| 10 | Broccoli | Green | 100.00 | 90.58 (13.27) | 57, 124, 37 |
| 11 | Cabbage | Green | 88.51 | 82 (18.65) | 81, 170, 64 |
| 12 | Cactus | Green | 99.33 | 80.07 (21.04) | 62, 145, 40 |
| 13 | Canary | Yellow | 88.28 | 83.22 (21.59) | 233, 231, 37 |
| 14 | Carrot | Orange | 97.35 | 91.69 (14.35) | 244, 123, 22 |
| 15 | Cauliflower | White | 88.67 | 75.86 (22.24) | 244, 245, 236 |
| 16 | Chalk | White | 96.58 | 84.92 (20.91) | 249, 248, 248 |
| 17 | Cheese | Yellow | 87.25 | 77.14 (19.78) | 234, 228, 72 |
| 18 | Cherry | Red | 89.26 | 89.15 (13.02) | 197, 22, 23 |
| 19 | Chick | Yellow | 95.21 | 84.28 (18.66) | 238, 233, 56 |
| 20 | Chocolate | Brown | 97.28 | 87.78 (15.27) | 110, 62, 24 |
| 21 | Cigar | Brown | 93.24 | 81.53 (21.34) | 113, 67, 30 |
| 22 | Clouds | White | 84.25 | 83.28 (18.38) | 245, 244, 244 |
| 23 | Coal | Black | 92.62 | 94.77 (10.44) | 6, 5, 5 |
| 24 | Cocoa | Brown | 96.62 | 85.62 (17.92) | 101, 55, 23 |
| 25 | Coffee | Brown | 76.35 | 86.31 (15.74) | 106, 60, 24 |
| 26 | Concrete | Grey | 94.67 | 86.38 (16.83) | 140, 137, 137 |
| 27 | Corn | Yellow | 97.26 | 84.66 (17.63) | 234, 227, 42 |
| 28 | Crocodile | Green | 82.24 | 78.94 (18.5) | 64, 108, 36 |
| 29 | Crow | Black | 98.66 | 91.53 (15.9) | 9, 7, 6 |
| 30 | Cucumber | Green | 100.00 | 85.9 (17.09) | 65, 136, 44 |
| 31 | Dirt | Brown | 77.70 | 80.46 (19.36) | 114, 66, 28 |
| 32 | Eggplant | Purple | 76.67 | 83.2 (18.45) | 95, 23, 116 |
| 33 | Elephant | Grey | 97.96 | 88.55 (14.83) | 135, 132, 131 |
| 34 | Field | Green | 95.20 | 77.55 (19.33) | 69, 171, 48 |
| 35 | Fire Engine | Red | 95.89 | 91.19 (11.74) | 231, 19, 14 |
| 36 | Flamingo | Pink | 96.64 | 89.6 (17.14) | 242, 123, 186 |
| 37 | Frog | Green | 96.69 | 77.4 (22.39) | 59, 142, 38 |
| 38 | Goldfish | Orange | 78.38 | 85.45 (14.66) | 240, 143, 19 |
| 39 | Grass | Green | 100.00 | 92.25 (10.75) | 54, 168, 37 |
| 40 | Heart | Red | 90.00 | 84.49 (18.16) | 213, 29, 24 |
| 41 | Hippopotamus | Grey | 89.04 | 79.52 (21.92) | 126, 122, 122 |
| 42 | Jeans | Blue | 97.32 | 81.1 (17.37) | 49, 77, 147 |
| 43 | Ketchup | Red | 100.00 | 91.44 (13.8) | 220, 20, 16 |
| 44 | Kiwi | Green | 89.93 | 77.92 (20.8) | 71, 164, 56 |
| 45 | Leaf | Green | 98.64 | 82.06 (17.66) | 54, 155, 39 |
| 46 | Lemon | Yellow | 98.67 | 95.33 (11.09) | 231, 231, 37 |
| 47 | Lettuce | Green | 99.32 | 87.43 (15.82) | 100, 201, 72 |
| 48 | Lime | Green | 96.00 | 88.29 (15.96) | 83, 207, 51 |
| 49 | Mud | Brown | 91.16 | 87.8 (15.12) | 97, 59, 25 |
| 50 | Mustard | Yellow | 95.27 | 85.99 (17.24) | 221, 200, 30 |
| 51 | Night | Black | 86.49 | 85.2 (15.83) | 23, 19, 23 |
| 52 | Ocean | Blue | 93.29 | 85.2 (16.68) | 54, 115, 189 |
| 53 | Orange | Orange | 100.00 | 98.19 (5.73) | 246, 132, 18 |
| 54 | Pea | Green | 98.67 | 90.89 (13.63) | 60, 169, 43 |
| 55 | Pig | Pink | 92.67 | 80.04 (18.64) | 221, 140, 180 |
| 56 | Pineapple | Yellow | 88.82 | 76.81 (21.33) | 232, 224, 49 |
| 57 | Plum | Purple | 87.84 | 80.96 (16.67) | 122, 32, 121 |
| 58 | Pool | Blue | 96.58 | 80.06 (20.31) | 76, 165, 224 |
| 59 | Poppy | Red | 92.05 | 89 (16) | 228, 23, 19 |
| 60 | Pumpkin | Orange | 94.00 | 89.91 (14.83) | 237, 139, 22 |
| 61 | Rhinoceros | Grey | 94.52 | 84.41 (17.75) | 134, 129, 128 |
| 62 | Salmon | Pink | 86.00 | 80.32 (17.37) | 239, 146, 167 |
| 63 | Sea | Blue | 89.19 | 81.28 (16.71) | 56, 125, 193 |
| 64 | Seal | Grey | 80.00 | 77.83 (21.66) | 137, 133, 132 |
| 65 | Shark | Grey | 81.08 | 78.98 (20.48) | 145, 143, 143 |
| 66 | Shrimp | Pink | 80.95 | 78.96 (21.7) | 239, 152, 179 |
| 67 | Sky | Blue | 99.32 | 83.72 (16.76) | 97, 178, 225 |
| 68 | Smoke | Grey | 80.14 | 75.35 (21.41) | 146, 143, 143 |
| 69 | Smurf | Blue | 93.29 | 87.5 (20.25) | 44, 124, 216 |
| 70 | Snow | White | 100.00 | 96.32 (8.41) | 249, 249, 249 |
| 71 | Spinach | Green | 99.32 | 91.31 (13.14) | 48, 117, 28 |
| 72 | Stop Sign | Red | 98.65 | 89.42 (15.25) | 236, 17, 13 |
| 73 | Strawberry | Red | 98.00 | 91.95 (10.12) | 231, 26, 21 |
| 74 | Sun | Yellow | 85.53 | 88.88 (14.64) | 239, 229, 33 |
| 75 | Swan | White | 96.62 | 91.67 (11.92) | 248, 246, 246 |
| 76 | Tomato | Red | 97.97 | 92.39 (11.27) | 229, 23, 17 |
| 77 | Tongue | Pink | 76.03 | 78.3 (21.03) | 221, 111, 141 |
| 78 | Yolk | Yellow | 80.27 | 86.38 (11.6) | 239, 215, 33 |
| 79 | Zucchini | Green | 82.35 | 80.29 (17.84) | 52, 117, 37 |

*Note.* This table presents the words included in the final dataset. Percentage of agreement refers to the proportion of participants who associated the word with the listed color. Mean association strength reflects participants' average rating of how strongly the word was associated with that color, with standard deviations presented in parentheses. RGB values provide the representative color values for the listed association. Items are numbered independently within this dataset table.

**Supplementary A2**

**Non-Dataset Words**

| **No.** | **Word** | **Associated Color** | **% Of Agreement** | **Mean Association Strength (SD)** | **RGB Values** |
| --- | --- | --- | --- | --- | --- |
| 1 | Apple | Green | 58.78 | 68.28 (20.34) | 75, 201, 60 |
| 2 | Apricot | Orange | 81.12 | 72.66 (22.39) | 236, 157, 54 |
| 3 | Artichoke | Green | 78.52 | 64.96 (27.25) | 91, 155, 66 |
| 4 | Asphalt | Black | 57.62 | 82.24 (23.87) | 28, 24, 22 |
| 5 | Basketball | Orange | 71.14 | 77.97 (22.73) | 230, 128, 23 |
| 6 | Bee | Yellow | 82.67 | 67.67 (19.32) | 231, 220, 37 |
| 7 | Beet | Purple | 63.51 | 79.88 (20.98) | 125, 31, 114 |
| 8 | Blueberries | Blue | 66.00 | 86.14 (17.25) | 48, 49, 139 |
| 9 | Bomb | Black | 71.43 | 70.68 (26.61) | 16, 14, 10 |
| 10 | Bread | Brown | 56.95 | 61.34 (23.99) | 161, 116, 59 |
| 11 | Camel | Brown | 74.15 | 70.66 (25.37) | 156, 112, 53 |
| 12 | Cancer | Black | 36.11 | 60.86 (32.32) | 11, 9, 9 |
| 13 | Cantaloupe | Yellow | 42.57 | 67.05 (22.89) | 222, 223, 78 |
| 14 | Cashew | Brown | 63.89 | 64.15 (26.25) | 183, 140, 75 |
| 15 | Caterpillar | Green | 84.25 | 69.98 (21.77) | 63, 170, 45 |
| 16 | Coconut | Brown | 55.33 | 74.4 (21.12) | 118, 71, 35 |
| 17 | Deer | Brown | 90.60 | 72.33 (23.92) | 134, 84, 36 |
| 18 | Dolphin | Grey | 75.00 | 81.51 (18.31) | 150, 149, 151 |
| 19 | Donkey | Grey | 59.73 | 72.62 (23.93) | 128, 124, 124 |
| 20 | Eagle | Brown | 62.42 | 64.14 (21.11) | 111, 65, 30 |
| 21 | Fire | Orange | 61.22 | 79.2 (17.19) | 239, 115, 22 |
| 22 | Fork | Grey | 91.28 | 60.12 (28.98) | 166, 163, 163 |
| 23 | Garlic | White | 85.14 | 70.73 (25.27) | 238, 235, 228 |
| 24 | Giraffe | Yellow | 44.44 | 63.92 (22.64) | 223, 200, 48 |
| 25 | Goat | White | 48.65 | 60.17 (28.6) | 235, 232, 230 |
| 26 | Grapefruit | Yellow | 44.44 | 73.28 (24.07) | 227, 218, 53 |
| 27 | Grapes | Green | 58.11 | 62.26 (21.11) | 108, 209, 87 |
| 28 | Hand Grenade | Green | 56.76 | 68.42 (24.58) | 60, 94, 34 |
| 29 | Iron | Grey | 72.30 | 76.38 (21.14) | 128, 125, 124 |
| 30 | Juice | Orange | 87.76 | 69.45 (23.93) | 242, 157, 31 |
| 31 | Lake | Blue | 88.59 | 74.93 (20.42) | 57, 113, 187 |
| 32 | Lava | Red | 48.99 | 78.07 (18.41) | 214, 38, 17 |
| 33 | Lion | Yellow | 44.14 | 64.81 (22.73) | 222, 197, 55 |
| 34 | Lips | Pink | 51.68 | 76.05 (19.93) | 231, 124, 151 |
| 35 | Lizard | Green | 82.55 | 65.67 (23.41) | 66, 147, 43 |
| 36 | Lobster | Red | 52.32 | 82.33 (16.74) | 213, 40, 31 |
| 37 | Mango | Orange | 52.78 | 69.79 (21.61) | 237, 161, 40 |
| 38 | Monkey | Brown | 83.67 | 73.29 (19.42) | 115, 70, 27 |
| 39 | Moon | White | 53.06 | 74.54 (23.53) | 239, 238, 235 |
| 40 | Mouse | Grey | 50.33 | 70.36 (23.87) | 153, 150, 150 |
| 41 | Mouth | Pink | 65.54 | 65.74 (24.06) | 231, 129, 159 |
| 42 | Nut | Brown | 91.95 | 74.26 (22.94) | 143, 92, 37 |
| 43 | Olive | Green | 81.08 | 73.28 (20.33) | 73, 133, 46 |
| 44 | Onion | White | 38.51 | 63.46 (24.37) | 242, 240, 231 |
| 45 | Peach | Orange | 59.73 | 66.75 (22.08) | 233, 153, 59 |
| 46 | Peanuts | Brown | 78.15 | 64.22 (24.83) | 168, 121, 61 |
| 47 | Pear | Green | 86.67 | 73.68 (21.86) | 116, 182, 77 |
| 48 | Pepper | Black | 48.99 | 83.49 (19.91) | 17, 16, 14 |
| 49 | Pickle | Green | 75.51 | 76.64 (20.28) | 66, 135, 38 |
| 50 | Potato | Brown | 63.95 | 68.03 (23.64) | 147, 104, 48 |
| 51 | Pretzels | Brown | 92.00 | 74.26 (24.53) | 145, 91, 38 |
| 52 | Radish | Red | 59.46 | 72.43 (24.06) | 220, 41, 45 |
| 53 | Rat | Grey | 57.82 | 76.04 (23.16) | 115, 111, 110 |
| 54 | Rose | Red | 63.95 | 78.97 (20.35) | 221, 18, 16 |
| 55 | Salad | Green | 99.33 | 70.47 (22.81) | 74, 189, 59 |
| 56 | Sand | Yellow | 63.70 | 76.99 (21.11) | 233, 219, 90 |
| 57 | Snail | Brown | 55.03 | 57.26 (25.65) | 127, 88, 44 |
| 58 | Tiger | Orange | 80.00 | 70.01 (20.79) | 232, 129, 25 |
| 59 | Tree | Green | 81.88 | 74.24 (17.08) | 60, 129, 35 |
| 60 | Turtle | Green | 64.67 | 73.78 (23.11) | 39, 76, 27 |
| 61 | Violin | Brown | 93.33 | 70.9 (25.62) | 118, 66, 21 |
| 62 | Water | Blue | 71.33 | 68.12 (26.37) | 116, 183, 229 |
| 63 | Watermelon | Green | 39.86 | 72.75 (20.76) | 60, 139, 38 |
| 64 | Whale | Grey | 49.32 | 72.32 (21.44) | 127, 124, 127 |

*Note.* This table presents words that were included in the broader stimulus pool but did not meet the dataset inclusion criteria. These words are retained for transparency and for possible use in future research. Percentage of agreement, mean association strength, and RGB values are reported in the same format as in Supplementary A1. Items are numbered independently within this non-dataset table.

**Supplementary B**

Supplementary B lists the previous studies that were used to construct the initial pool of color-associated words for Experiment 1. The purpose of this section is to make the stimulus-selection process transparent and to document the literature base from which the candidate words were extracted. The search was literature-based and structured, but it was not intended to serve as a formal systematic review. Rather, the goal was to identify a broad and relevant set of studies from cognitive science and related areas that used color-associated words, object-color associations, memory colors, semantic Stroop stimuli, or similar color-word materials.
 The literature-based search procedure used to construct the initial stimulus pool involved several steps. **Step 1** consisted of a Google Scholar search using the terms **“semantic Stroop,” “color-associated words,” “color diagnosticity,” “memory color,” “object-color associations,”** and **“color-word associations.”** These terms were selected because they directly target studies using color-associated words, object-color knowledge, memory colors, color-diagnostic stimuli, and semantic Stroop materials. **Step 2** involved identifying an initial set of relevant studies from the search results. Studies were considered relevant when they included color-associated words, object-color associations, memory-color stimuli, semantic Stroop materials, or related color-word experimental stimuli. **Step 3** involved citation-chaining from this initial set of studies. Specifically, we examined the reference lists of the identified papers to locate earlier studies that may have used similar stimuli or provided relevant theoretical and methodological background. We also examined papers that cited the identified studies, allowing us to locate more recent studies that used related color-associated materials. **Step 4** consisted of a second round of citation-chaining, in which the same procedure was repeated for the newly identified studies. This second round was used to broaden the literature pool and reduce the likelihood that relevant studies using color-associated stimuli were missed. **Step 5** involved manual screening of all candidate studies. During this stage, studies were retained only when they used concrete color-associated object or concept words as experimental stimuli. This criterion was important because the goal of the dataset was to identify words that naturally and reliably evoke specific colors, such as object words with strong color associations, rather than words with only abstract, metaphorical, or emotional color meanings. **Step 6** involved excluding studies that were outside the scope of the present dataset. Studies were excluded when they focused primarily on abstract color meanings, affective color associations, or materials that were not relevant to cognitive color-word research. **Step 7** involved extracting the relevant stimuli from the retained studies. This procedure resulted in a final literature pool of **124 studies**, from which **143 candidate color-associated words** were extracted and included in **Experiment 1**. The full literature-based selection procedure is summarized visually in Figure S1.

**Figure S1
*Literature-Based Procedure Used to Identify the Initial Pool of Color-Associated Words***

***Note.*** The figure summarizes the structured literature-based procedure used to identify the initial pool of color-associated words. Google Scholar searches were followed by two rounds of citation-chaining and manual screening of candidate studies. Studies were retained if they used concrete color-associated object or concept words as experimental stimuli and were excluded if they focused mainly on abstract color meanings, affective color associations, or materials outside the scope of cognitive color-word research. This procedure resulted in 124 retained studies, from which 143 candidate color-associated words were extracted for Experiment 1.

***List of Papers that Used Color-associated Stimuli***

1. Anderson, S. E., Chiu, E., Huette, S., & Spivey, M. J. (2011). On the temporal dynamics of language-mediated vision and vision-mediated language. Acta Psychologica, 137(2), 181-189.

2. Anton, K. F. (2016). Examining Activation of Lexical and Semantic Representations Without Intention: Evidence from Event-Related Potentials.

3. Anton, K. F., Gould, L., & Borowsky, R. (2014). Activation of lexical and semantic representations without intention along GPC-sublexical and orthographic-lexical reading pathways in a Stroop paradigm. Journal of Experimental Psychology: Learning, Memory, and Cognition, 40(3), 623.

4. Augustinova, M., & Ferrand, L. (2012). Suggestion does not de-automatize word reading: Evidence from the semantically based Stroop task. Psychonomic Bulletin & Review, 19, 521-527.

5. Augustinova, M., & Ferrand, L. (2012). The influence of mere social presence on Stroop interference: New evidence from the semantically-based Stroop task. Journal of Experimental Social Psychology, 48(5), 1213-1216.

6. Augustinova, M., & Ferrand, L. (2014). Automaticity of word reading: Evidence from the semantic Stroop paradigm. Current Directions in Psychological Science, 23(5), 343-348.

7. Augustinova, M., & Ferrand, L. (2014). Social priming of dyslexia and reduction of the Stroop effect: What component of the Stroop effect is actually reduced?. Cognition, 130(3), 442-454.

8. Augustinova, M., Clarys, D., Spatola, N., & Ferrand, L. (2018). Some further clarifications on age-related differences in Stroop interference. Psychonomic Bulletin & Review, 25, 767-774.

9. Augustinova, M., Flaudias, V., & Ferrand, L. (2010). Single-letter coloring and spatial cuing do not eliminate or reduce a semantic contribution to the Stroop effect. Psychonomic Bulletin & Review, 17, 827-833.

10. Augustinova, M., Parris, B. A., & Ferrand, L. (2019). The loci of Stroop interference and facilitation effects with manual and vocal responses. Frontiers in psychology, 10, 1786.

11. Augustinova, M., Silvert, L., Ferrand, L., Llorca, P. M., & Flaudias, V. (2015). Behavioral and electrophysiological investigation of semantic and response conflict in the Stroop task. Psychonomic Bulletin & Review, 22, 543-549.

12. Augustinova, M., Silvert, L., Spatola, N., & Ferrand, L. (2018). Further investigation of distinct components of Stroop interference and of their reduction by short response-stimulus intervals. Acta Psychologica, 189, 54-62.

13. Bartleson, C. J. (1960). Memory colors of familiar objects. Journal of the Optical Society of America, 50(1), 73-77. https://doi.org/10.1364/JOSA.50.000073

14. Besner, D., & Reynolds, M. (2017). Is semantic activation from print capacity limited? Evidence from the psychological refractory period paradigm. Psychonomic Bulletin & Review, 24, 907-913.

15. Besner, D., Risko, E. F., Stolz, J. A., White, D., Reynolds, M., O’Malley, S., & Robidoux, S. (2016). Varieties of attention: Their roles in visual word identification. Current Directions in Psychological Science, 25(3), 162-168.

16. Bhangal, S., Cho, H., Geisler, M. W., & Morsella, E. (2016). The prospective nature of voluntary action: Insights from the reflexive imagery task. Review of General Psychology, 20(1), 101-117.

17. Bidet-Ildei, C., Meugnot, A., Beauprez, S. A., Gimenes, M., & Toussaint, L. (2017). Short-term upper limb immobilization affects action-word understanding. Journal of Experimental Psychology: Learning, Memory, and Cognition, 43(7), 1129.

18. Bramão, I., Faísca, L., Petersson, K. M., & Reis, A. (2010). The influence of surface color information and color knowledge information in object recognition. The American Journal of Psychology, 123(4), 437-446.

19. Bramão, I., Reis, A., Petersson, K. M., & Faísca, L. (2011). The role of color information on object recognition: A review and meta-analysis. Acta psychologica, 138(1), 244-253.

20. Bramão, I., Reis, A., Petersson, K. M., & Faísca, L. (2016). Knowing that strawberries are red and seeing red strawberries: the interaction between surface colour and colour knowledge information. Journal of Cognitive Psychology, 28(6), 641-657.

21. Brodeur, M. B., O’Sullivan, M., & Crone, L. (2017). The impact of image format and normative variables on episodic memory. Cogent Psychology, 4(1), 1328869.

22. Brunel, L., Servajean, P., Heurley, L., & Vermeulen, N. (2017). Does banana spontaneously activate yellow color? Color-related concepts help withcolor discrimination. In Proceedings of the Annual Meeting of the Cognitive Science Society (Vol. 39).

23. Burt, J. S. (1994). Identity primes produce facilitation in a colour naming task. The Quarterly Journal of Experimental Psychology, 47(4), 957-1000.

24. Burt, J. S. (2002). Why do non-color words interfere with color naming?. Journal of Experimental Psychology: Human Perception and Performance, 28(5), 1019.

25. Bush, G., Whalen, P. J., Rosen, B. R., Jenike, M. A., McInerney, S. C., & Rauch, S. L. (1998). The counting Stroop: an interference task specialized for functional neuroimaging—validation study with functional MRI. Human brain mapping, 6(4), 270-282.

26. Chang, S., Lewis, D. E., & Pearson, J. (2013). The functional effects of color perception and color imagery. Journal of Vision, 13(10), 4-4.

27. Connell, L., & Lynott, D. (2009). Is a bear white in the woods? Parallel representation of implied object color during language comprehension. Psychonomic Bulletin & Review, 16, 573-577.

28. Cortese, M. J., Khanna, M. M., & Von Nordheim, D. (2019). Incidental memory for colour word associates processed in colour naming and reading aloud tasks: is a blue ocean more memorable than a yellow one?. Memory, 27(7), 924-930.

29. Cortese, M. J., Toppi, S., Khanna, M. M., & Santo, J. B. (2020). AoA effects in reading aloud and lexical decision: Locating the (semantic) locus in terms of the number of backward semantic associations. Quarterly Journal of Experimental Psychology, 73(11), 2036-2044.

30. Cox, W. M., Fadardi, J. S., & Pothos, E. M. (2006). The addiction-stroop test: Theoretical considerations and procedural recommendations. Psychological bulletin, 132(3), 443.

31. Cummine, J., Aalto, D., Ostevik, A., Cheema, K., & Hodgetts, W. (2018). “To Name or Not to Name: That is the Question”: The Role of Response Inhibition in Reading. Journal of psycholinguistic research, 47, 999-1014.

32. Cummine, J., Cullum, A., Aalto, D., Sereda, T., Fleming, C., Reed, A., ... & Hodgetts, W. E. (2021). From lollipops to lidocaine: The need for a universal print-to-speech framework. Canadian Journal of Experimental Psychology/Revue canadienne de psychologie expérimentale, 75(3), 279.

33. Dalrymple-Alford, E. C. (1972). Associative facilitation and interference in the Stroop color-word task. Perception & Psychophysics, 11, 274-276.

34. De Houwer, J., & d'Ydewalle, G. (1994). Stroop-like interference in sorting for intrinsic color: a test of the Glaser and Glaser (1989) model. Acta psychologica, 85(2), 123-137.

35. De Marchis, G., Rivero Expósito, M. D. P., & Reales Avilés, J. M. (2013). Psychological distance and reaction time in a Stroop task. Cognitive processing, 14, 401-410.

36. Dhooge, E., & Hartsuiker, R. J. (2010). The distractor frequency effect in picture–word interference: Evidence for response exclusion. Journal of Experimental Psychology: Learning, Memory, and Cognition, 36(4), 878.

37. Dyer, F. N. (1973). The Stroop phenomenon and its use in the study of perceptual, cognitive, and response processes. Memory & Cognition, 1(2), 106-120.

38. Entel, O., & Tzelgov, J. (2018). Focusing on task conflict in the Stroop effect. Psychological research, 82, 284-295.

39. Ferrand, L., & Augustinova, M. (2014). Differential effects of viewing positions on standard versus semantic Stroop interference. Psychonomic bulletin & review, 21, 425-431.

40. Ferrand, L., Ducrot, S., Chausse, P., Maïonchi‐Pino, N., O’Connor, R. J., Parris, B. A., Perret, P., Riggs, K. J., & Augustinova, M. (2020). Stroop interference is a composite phenomenon: Evidence from distinct developmental trajectories of its components. Developmental Science, 23(2), e12899.

41. Finkbeiner, M., & Caramazza, A. (2008). Modulating the masked congruence priming effect with the hands and the mouth. Journal of Experimental Psychology: Human Perception and Performance, 34(4), 894.

42. Finkbeiner, M., & Palermo, R. (2009). The role of spatial attention in nonconscious processing: A comparison of face and nonface stimuli. Psychological Science, 20(1), 42-51.

43. Finkbeiner, M., Song, J. H., Nakayama, K., & Caramazza, A. (2008). Engaging the motor system with masked orthographic primes: A kinematic analysis. Visual cognition, 16(1), 11-22.

44. Flaisch, T., Imhof, M., Schmälzle, R., Wentz, K. U., Ibach, B., & Schupp, H. T. (2015). Implicit and explicit attention to pictures and words: an fMRI-study of concurrent emotional stimulus processing. Frontiers in Psychology, 6, 1861.

45. Fox, L. A., Shor, R. E., & Steinman, R. J. (1971). Semantic gradients and interference in naming color, spatial direction, and numerosity. Journal of Experimental Psychology, 91(1), 59.

46. Glaser, W. R., & Glaser, M. (1993). Colors as properties: Stroop-like effects between objects and their colors. In Advances in psychology (Vol. 101, pp. 1-32). North-Holland.

47. Glaser, W. R., & Glaser, M. O. (1989). Context effects in stroop-like word and picture processing. Journal of Experimental Psychology: General, 118(1), 13.

48. Goldfarb, L., & Tzelgov, J. (2007). The cause of the within-language Stroop superiority effect and its implications. Quarterly Journal of Experimental Psychology, 60(2), 179-185.

49. Gould, L., Mickleborough, M. J., Ekstrand, C., Lorentz, E., & Borowsky, R. (2017). Examining the neuroanatomical and the behavioural basis of the effect of basic rhythm on reading aloud. Language, cognition and neuroscience, 32(6), 724-742.

50. Gould, L., Mickleborough, M. J., Lorentz, E., Ekstrand, C., & Borowsky, R. (2018). A behavioral and fMRI examination of the effect of rhythm on reading noun-verb homographs aloud. Language, cognition and neuroscience, 33(7), 829-849.

51. Harrison, N. S., & Boese, E. (1976). The locus of semantic interference in the “Stroop” color-naming task. Perception & Psychophysics, 20, 408-412.

52. Heurley, L. (2012). Mémoire et perception: l'influence de la simulation de la couleur sur la perception de la couleur (Doctoral dissertation, Université Paul Valéry-Montpellier III).

53. Heurley, L. P., Brouillet, T., Chesnoy, G., & Brouillet, D. (2013). Color perception involves color representations firstly at a semantic level and then at a lexical level. Cognitive processing, 14(1), 19-29.

54. Heurley, L., & Vermeulen, N. (2017). Does banana spontaneously activate yellow color? Color-related concepts help with color discrimination.

55. Holle, C., Neely, J. H., & Heimberg, R. G. (1997). The effects of blocked versus random presentation and semantic relatedness of stimulus words on response to a modified Stroop task among social phobics. Cognitive therapy and research, 21, 681-697.

56. Hsu, N. S., Schlichting, M. L., & Thompson-Schill, S. L. (2014). Feature diagnosticity affects representations of novel and familiar objects. Journal of Cognitive Neuroscience, 26(12), 2735-2749. https://doi.org/10.1162/jocn_a_00661

57. Jiang, J., Zhang, Q., & Van Gaal, S. (2015). EEG neural oscillatory dynamics reveal semantic and response conflict at difference levels of conflict awareness. Scientific reports, 5(1), 12008.

58. Joseph, J. E., & Proffitt, D. R. (1996). Semantic versus perceptual influences of color in object recognition. Journal of Experimental Psychology: Learning, Memory, and Cognition, 22(2), 407.

59. Kemmerer, D. (2015). Are the motor features of verb meanings represented in the precentral motor cortices? Yes, but within the context of a flexible, multilevel architecture for conceptual knowledge. Psychonomic Bulletin & Review, 22, 1068-1075.

60. Kinoshita, S., Mills, L., & Norris, D. (2018). The semantic Stroop effect is controlled by endogenous attention. Journal of Experimental Psychology: Learning, Memory, and Cognition, 44(11), 1730.

61. Klein, G. S. (1964). Semantic power measured through the interference of words with color-naming. The American journal of psychology, 77(4), 576-588.

62. Labuschagne, E. M., & Besner, D. (2015). Automaticity revisited: When print doesn't activate semantics. Frontiers in Psychology, 6, 117.

63. Lachter, J., Ruthruff, E., Lien, M. C., & Mccann, R. S. (2008). Is attention needed for word identification? Evidence from the Stroop paradigm. Psychonomic Bulletin & Review, 15, 950-955.

64. Landry, M., Lifshitz, M., & Raz, A. (2017). Brain correlates of hypnosis: A systematic review and meta-analytic exploration. Neuroscience & Biobehavioral Reviews, 81, 75-98.

65. Levin, Y., & Tzelgov, J. (2016). Contingency learning is not affected by conflict experience: Evidence from a task conflict-free, item-specific Stroop paradigm. Acta psychologica, 164, 39-45.

66. Levin, Y., & Tzelgov, J. (2016). What Klein’s “semantic gradient” does and does not really show: Decomposing Stroop interference into task and informational conflict components. Frontiers in Psychology, 7, 249.

67. Liefooghe, B., Hughes, S., Schmidt, J. R., & De Houwer, J. (2020). Stroop-like effects of derived stimulus–stimulus relations. Journal of Experimental Psychology: Learning, Memory, and Cognition, 46(2), 327.

68. Lifshitz, M., Bonn, N. A., Fischer, A., Kashem, I. F., & Raz, A. (2013). Using suggestion to modulate automatic processes: From Stroop to McGurk and beyond. Cortex, 49(2), 463-473.

69. Lloyd-Jones, T. J., & Nakabayashi, K. (2009). Independent effects of colour on object identification and memory. Quarterly journal of experimental psychology, 62(2), 310-322.

70. Logan, G. D. (1980). Attention and automaticity in Stroop and priming tasks: Theory and data. Cognitive psychology, 12(4), 523-553.

71. Lorentz, E. J. (2016). Literally and figuratively speaking: How concepts and perception influence each other using Stroop paradigms (Doctoral dissertation, University of Saskatchewan).

72. Lorentz, E., Gould, L., Mickleborough, M., Ekstrand, C., Boyer, M., Cheesman, J., & Borowsky, R. (2015). All in one fell Stroop: Examining consciousness thresholds with a multiple response paradigm. Psychology of Consciousness: Theory, Research, and Practice, 2(2), 111.

73. Lorentz, E., McKibben, T., Ekstrand, C., Gould, L., Anton, K., & Borowsky, R. (2016). Disentangling genuine semantic Stroop effects in reading from contingency effects: On the need for two neutral baselines. Frontiers in Psychology, 7, 386.

74. Lu, C. H., & Proctor, R. W. (1995). The influence of irrelevant location information on performance: A review of the Simon and spatial Stroop effects. Psychonomic bulletin & review, 2, 174-207.

75. Magalhães De Saldanha da Gama, P. A., Slama, H., Caspar, E. A., Gevers, W., & Cleeremans, A. (2013). Placebo-suggestion modulates conflict resolution in the Stroop Task. PLoS One, 8(10), e75701.

76. Mahon, B. Z., Costa, A., Peterson, R., Vargas, K. A., & Caramazza, A. (2007). Lexical selection is not by competition: a reinterpretation of semantic interference and facilitation effects in the picture-word interference paradigm. Journal of Experimental Psychology: Learning, Memory, and Cognition, 33(3), 503.

77. Majeres, R. L. (1974). The combined effects of stimulus and response conditions on the delay in identifying the print color of words.

78. Mani, N., Johnson, E., McQueen, J. M., & Huettig, F. (2013). How yellow is your banana? Toddlers' language-mediated visual search in referent-present tasks. Developmental Psychology, 49(6), 1036.

79. Manwell, L. A., Roberts, M. A., & Besner, D. (2004). Single letter coloring and spatial cuing eliminates a semantic contribution to the Stroop effect. Psychonomic Bulletin & Review, 11(3), 458-462.

80. Marangolo, P., Di Pace, E., & Pizzamiglio, L. (1993). Priming effect in a color discrimination task. Perceptual and motor skills, 77(1), 259-269.

81. McClain, L. (1983). Color priming affects Stroop interference. Perceptual and Motor Skills, 56(2), 643-651.

82. McKenna, F. P., & Sharma, D. (1995). Intrusive cognitions: an investigation of the emotional Stroop task. Journal of Experimental Psychology: learning, memory, and cognition, 21(6), 1595.

83. Mitterer, H., Horschig, J. M., Müsseler, J., & Majid, A. (2009). The influence of memory on perception: It’s not what things look like, it’s what you call them. Journal of Experimental Psychology: Learning, Memory, and Cognition, 35(6), 1557-1562. https://doi.org/10.1037/a0017019

84. Morita, A., & Kambara, T. (2022). Color bizarreness effects in object memory: Evidence from a recall test and eye tracking. Color Research & Application, 47(1), 55-64.

85. Morsella, E., Godwin, C. A., Jantz, T. K., Krieger, S. C., & Gazzaley, A. (2016). Homing in on consciousness in the nervous system: An action-based synthesis. Behavioral and Brain Sciences, 39, e168.

86. Mädebach, A., Oppermann, F., Hantsch, A., Curda, C., & Jescheniak, J. D. (2011). Is there semantic interference in delayed naming?.

87. Ménard-Buteau, C., & Cavanagh, P. (1984). Localisation de l'interférence forme/couleur au niveau perceptuel dans une tâche de type Stroop avec des stimuli-dessins. Canadian Journal of Psychology/Revue canadienne de psychologie, 38(3), 421.

88. Naor-Raz, G., Tarr, M. J., & Kersten, D. (2003). Is color an intrinsic property of object representation? Perception, 32(6), 667-680. https://doi.org/10.1068/p5050

89. Nijboer, T. C., van Zandvoort, M. J., & de Haan, E. H. (2006). Seeing red primes tomato: Evidence for comparable priming from colour and colour name primes to semantically related word targets. Cognitive processing, 7, 269-274.

90. Parris, B. A., Wadsley, M. G., Hasshim, N., Benattayallah, A., Augustinova, M., & Ferrand, L. (2019). An fMRI study of response and semantic conflict in the Stroop task. Frontiers in Psychology, 10, 2426.

91. Redmann, A., FitzPatrick, I., & Indefrey, P. (2019). The time course of colour congruency effects in picture naming. Acta Psychologica, 196, 96-108.

92. Regan, J. (1978). Involuntary automatic processing in color-naming tasks. Perception & Psychophysics, 24, 130-136.

93. Risko, E. F., Schmidt, J. R., & Besner, D. (2006). Filling a gap in the semantic gradient: Color associates and response set effects in the Stroop task. Psychonomic Bulletin & Review, 13(2), 310-315.

94. Roelofs, A., Piai, V., & Schriefers, H. (2011). Selective attention and distractor frequency in naming performance: Comment on Dhooge and Hartsuiker (2010).

95. Roux, S., & Bonin, P. (2016). “RED” matters when naming “CAR”: The cascading activation of nontarget properties. Journal of experimental psychology: learning, memory, and cognition, 42(3), 475.

96. Scaltritti, M., Job, R., & Sulpizio, S. (2022). Different types of semantic interference, same lapses of attention: Evidence from Stroop tasks. Memory & Cognition, 50(5), 898-910.

97. Scheibe, K. E., Shaver, P. R., & Carrier, S. C. (1967). Color association values and response interference on variants of the Stroop test. Acta psychologica, 26, 286-295.

98. Schmidt, J. R., & Cheesman, J. (2005). Dissociating stimulus-stimulus and response-response effects in the Stroop task. Canadian Journal of Experimental Psychology/Revue canadienne de psychologie expérimentale, 59(2), 132.

99. Schmidt, J. R., Cheesman, J., & Besner, D. (2013). You can’t Stroop a lexical decision: Is semantic processing fundamentally facilitative?. Canadian Journal of Experimental Psychology/Revue canadienne de psychologie expérimentale, 67(2), 130.

100. Schmidt, J. R., Hartsuiker, R. J., & De Houwer, J. (2018). Interference in Dutch–French Bilinguals. Experimental psychology.

101. Scorolli, C., & Borghi, A. M. (2015). Square bananas, blue horses: the relative weight of shape and color in concept recognition and representation. Frontiers in Psychology, 6, 1542.

102. Seifert, L. S., & Johnson, N. F. (1994). On the naming of color words and color patches. Memory & cognition, 22(2), 169-180.

103. Sharma, D., & McKenna, F. P. (1998). Differential components of the manual and vocal Stroop tasks. Memory & Cognition, 26, 1033-1040.

104. Shichel, I., & Tzelgov, J. (2018). Modulation of conflicts in the Stroop effect. Acta Psychologica, 189, 93-102.

105. Spatola, N., & Huguet, P. (2021). Cognitive impact of anthropomorphized robot gaze: anthropomorphic gaze as social cues. ACM Transactions on Human-Robot Interaction (THRI), 10(4), 1-14.

106. Spatola, N., Belletier, C., Chausse, P., Augustinova, M., Normand, A., Barra, V., ... & Huguet, P. (2019). Improved cognitive control in presence of anthropomorphized robots. International Journal of Social Robotics, 11, 463-476.

107. Sulpizio, S., Job, R., Leoni, P., & Scaltritti, M. (2022). Prepotent task-irrelevant semantic information is dampened by domain-specific control mechanisms during visual word recognition. Quarterly Journal of Experimental Psychology, 75(3), 390-405.

108. Sulpizio, S., Spinelli, G., & Scaltritti, M. (2024). Semantic Stroop interference is modulated by the availability of executive resources: Insights from delta-plot analyses and cognitive load manipulation. Memory & Cognition, 52(6), 1422-1438.

109. Tanaka, J. W., & Presnell, L. M. (1999). Color diagnosticity in object recognition. Perception & Psychophysics, 61(6), 1140-1153.

110. Terhune, D. B., Cleeremans, A., Raz, A., & Lynn, S. J. (2017). Hypnosis and top-down regulation of consciousness. Neuroscience & Biobehavioral Reviews, 81, 59-74.

111. The effect of high-frequency rTMS of the left dorsolateral prefrontal cortex on the resolution of response, semantic and task conflict in the colour-word Stroop task

112. Therriault, D. J., Yaxley, R. H., & Zwaan, R. A. (2009). The role of color diagnosticity in object recognition and representation. Cognitive processing, 10, 335-342.

113. van Veen, V., & Carter, C. S. (2005). Separating semantic conflict and response conflict in the Stroop task: a functional MRI study. Neuroimage, 27(3), 497-504.

114. Vinerte, S. (2018). Effects of Bilingualism on Cognitive Control: Considering the Age of Immersion and Different Linguistic Environments (Doctoral dissertation, Université d'Ottawa/University of Ottawa).

115. Wadsley, M. (2019). An fMRI and TMS investigation of response, semantic, and task conflict in the Stroop task (Doctoral dissertation, Bournemouth University).

116. Wang, K., Mecklinger, A., Hofmann, J., & Weng, X. (2010). From orthography to meaning: an electrophysiological investigation of the role of phonology in accessing meaning of Chinese single-character words. Neuroscience, 165(1), 101-106.

117. Wentura, D. (1999). Activation and inhibition of affective information: For negative priming in the evaluation task. Cognition & Emotion, 13(1), 65-91.

118. White, D. (2017). Is Semantic Activation from Print Automatic? An Investigation Using the Psychological Refractory Period and Task Set Paradigms.

119. White, D., Risko, E. F., & Besner, D. (2016). The semantic Stroop effect: An ex-Gaussian analysis. Psychonomic bulletin & review, 23, 1576-1581.

120. Witzel, C., Valkova, H., Hansen, T., & Gegenfurtner, K. R. (2011). Object knowledge modulates colour appearance. i-Perception, 2(1), 13-49. https://doi.org/10.1068/i0396

121. Yee, E., Ahmed, S. Z., & Thompson-Schill, S. L. (2012). Colorless green ideas (can) prime furiously. Psychological Science, 23(4), 364-369.

122. Yendrikhovskij, S. N., Blommaert, F. J. J., & de Ridder, H. (1999). Representation of memory prototype for an object color. Color Research & Application, 24(6), 393-410. https://doi.org/10.1002/(SICI)1520-6378(199912)24:6<393::AID-COL3>3.0.CO;2-Z

123. Zhou, P., Wang, K., Zhang, C., & Ren, P. (2019). Attention effects on the processing of task-relevant and task-irrelevant conflict. In Advances in Computational Science and Computing (pp. 453-459). Springer International Publishing.

124. Zhou, W., Deng, L., & Ding, J. (2021). Neural mechanism underlying the effects of object color on episodic memory. Acta Psychologica Sinica, 53(3), 229.

**Supplementary C** Supplementary C provides additional item-level information about the second and third most frequently reported color associations for the words included in the stimulus pool. Whereas Supplementary A presents the most strongly associated color for each word, Supplementary C is intended to show whether a word also elicited additional meaningful color associations. This information is important because many objects and concepts are not associated with only one color. For example, some words may have a clear dominant color association but may also be linked to another common color. These secondary and tertiary associations can be useful for researchers who wish to construct carefully controlled congruent and incongruent stimulus pairings. This section is especially relevant for studies using semantic Stroop, color-naming, object-recognition, or memory-color paradigms. In such studies, an intended “incongruent” color may not be fully unrelated to the word if that color was also reported as a second or third association by participants. Thus, Supplementary C allows researchers to identify possible alternative associations and avoid stimulus pairings that may unintentionally weaken the contrast between congruent and incongruent trials. The information is divided into two parts: Supplementary C1 reports the second most frequently associated color for relevant words, and Supplementary C2 reports the third most frequently associated color for words that elicited an additional association.

**Supplementary C1**

***Second Most Frequently Associated Colors***

| **No.** | **Word** | **Associated Color** | **% Of Agreement** | **Mean Association Strength** | **RGB Values** |
| --- | --- | --- | --- | --- | --- |
| **Dataset words** | | | | | |
| 4 | Bear | Black | 12.93 | 60.84 | 8, 8, 8 |
| 6 | Bone | Grey | 6.62 | 70.30 | 208, 204, 204 |
| 11 | Cabbage | White | 7.43 | 60.64 | 230, 235, 224 |
| 15 | Cauliflower | Green | 6.00 | 47.11 | 122, 197, 112 |
| 17 | Cheese | Orange | 8.05 | 73.43 | 230, 141, 43 |
| 18 | Cherry | Purple | 6.04 | 71.00 | 134, 27, 97 |
| 22 | Clouds | Grey | 13.70 | 67.00 | 184, 184, 184 |
| 25 | Coffee | Black | 22.97 | 81.12 | 27, 18, 14 |
| 28 | Crocodile | Grey | 9.87 | 64.87 | 113, 113, 109 |
| 31 | Dirt | Black | 12.84 | 72.84 | 47, 40, 39 |
| 32 | Eggplant | Green | 12.67 | 64.00 | 65, 140, 42 |
| 38 | Goldfish | Yellow | 18.92 | 79.64 | 228, 209, 27 |
| 40 | Heart | Pink | 7.33 | 78.45 | 219, 85, 110 |
| 44 | Kiwi | Brown | 8.05 | 71.67 | 152, 108, 39 |
| 51 | Night | Blue | 9.46 | 73.17 | 42, 32, 118 |
| 56 | Pineapple | Orange | 5.26 | 58.00 | 227, 162, 51 |
| 57 | Plum | Red | 9.46 | 78.50 | 165, 28, 25 |
| 62 | Salmon | Orange | 8.67 | 77.62 | 233, 136, 72 |
| 64 | Seal | Black | 9.66 | 67.00 | 28, 27, 27 |
| 65 | Shark | Blue | 12.16 | 81.83 | 66, 93, 141 |
| 66 | Shrimp | Orange | 8.16 | 71.25 | 228, 131, 41 |
| 68 | Smoke | Black | 12.33 | 81.67 | 38, 36, 36 |
| 74 | Sun | Orange | 13.82 | 82.19 | 239, 145, 33 |
| 77 | Tongue | Red | 20.55 | 77.37 | 208, 54, 56 |
| 78 | Yolk | Orange | 19.73 | 90.01 | 244, 161, 26 |
| 79 | Zucchini | Purple | 7.19 | 79.18 | 108, 34, 122 |
| **Non-dataset words** | | | | | |
| 1 | Apple | Red | 37.84 | 71.79 | 218, 24, 22 |
| 2 | Apricot | Yellow | 11.89 | 62.18 | 230, 189, 82 |
| 3 | Artichoke | Purple | 6.71 | 58.50 | 138, 103, 166 |
| 4 | Asphalt | Grey | 34.44 | 78.98 | 109, 106, 106 |
| 5 | Basketball | Brown | 13.42 | 76.32 | 141, 77, 16 |
| 6 | Bee | Black | 10.67 | 52.56 | 35, 25, 20 |
| 7 | Beet | Red | 31.08 | 80.13 | 170, 21, 25 |
| 8 | Blueberries | Purple | 27.33 | 77.61 | 79, 35, 120 |
| 9 | Bomb | Grey | 13.61 | 68.19 | 94, 94, 93 |
| 10 | Bread | White | 41.06 | 67.52 | 243, 239, 228 |
| 11 | Camel | Yellow | 17.01 | 61.00 | 220, 209, 82 |
| 12 | Cancer | Pink | 18.75 | 35.96 | 232, 116, 168 |
| 13 | Cantaloupe | Orange | 24.32 | 73.53 | 245, 166, 58 |
| 14 | Cashew | Yellow | 20.14 | 48.03 | 227, 221, 122 |
| 16 | Coconut | White | 42.67 | 71.63 | 248, 247, 246 |
| 18 | Dolphin | Blue | 19.59 | 77.14 | 89, 140, 193 |
| 19 | Donkey | Brown | 38.26 | 71.00 | 125, 83, 46 |
| 20 | Eagle | Grey | 12.75 | 64.74 | 120, 118, 118 |
| 21 | Fire | Red | 28.57 | 80.66 | 225, 27, 22 |
| 22 | Fork | White | 7.38 | 30.18 | 229, 226, 227 |
| 23 | Garlic | Grey | 5.41 | 55.38 | 174, 174, 174 |
| 24 | Giraffe | Orange | 28.47 | 63.20 | 220, 138, 40 |
| 25 | Goat | Brown | 25.00 | 56.33 | 143, 96, 59 |
| 26 | Grapefruit | Pink | 32.68 | 65.59 | 235, 108, 160 |
| 27 | Grapes | Purple | 30.41 | 71.40 | 120, 42, 122 |
| 28 | Hand Grenade | Grey | 21.62 | 72.66 | 104, 101, 101 |
| 29 | Iron | Black | 12.84 | 68.68 | 33, 31, 21 |
| 30 | Juice | Yellow | 7.48 | 70.64 | 242, 215, 69 |
| 31 | Lake | Green | 6.04 | 50.33 | 63, 122, 78 |
| 32 | Lava | Orange | 38.93 | 79.31 | 233, 110, 24 |
| 33 | Lion | Brown | 30.34 | 61.16 | 158, 113, 41 |
| 34 | Lips | Red | 48.32 | 79.63 | 227, 53, 49 |
| 35 | Lizard | Brown | 8.72 | 58.69 | 147, 109, 58 |
| 36 | Lobster | Pink | 26.49 | 75.58 | 233, 121, 132 |
| 37 | Mango | Yellow | 29.86 | 71.86 | 237, 210, 32 |
| 38 | Monkey | Grey | 10.88 | 71.56 | 130, 127, 126 |
| 39 | Moon | Grey | 31.29 | 76.78 | 170, 167, 167 |
| 40 | Mouse | Brown | 32.45 | 71.16 | 118, 71, 28 |
| 41 | Mouth | Red | 32.43 | 68.33 | 213, 58, 58 |
| 43 | Olive | Black | 16.22 | 78.58 | 11, 9, 9 |
| 44 | Onion | Brown | 32.43 | 75.52 | 193, 157, 132 |
| 45 | Peach | Pink | 28.19 | 64.24 | 231, 140, 141 |
| 46 | Peanuts | Yellow | 11.92 | 54.83 | 219, 198, 91 |
| 47 | Pear | Yellow | 10.00 | 71.93 | 228, 214, 29 |
| 48 | Pepper | Red | 38.26 | 72.40 | 221, 19, 17 |
| 49 | Pickle | Brown | 10.88 | 61.81 | 115, 78, 37 |
| 50 | Potato | White | 23.13 | 76.32 | 235, 230, 216 |
| 52 | Radish | Pink | 24.32 | 76.33 | 221, 61, 125 |
| 53 | Rat | Brown | 29.93 | 77.86 | 104, 64, 29 |
| 54 | Rose | Pink | 29.93 | 73.36 | 236, 124, 175 |
| 56 | Sand | Brown | 15.07 | 66.36 | 181, 141, 76 |
| 57 | Snail | Grey | 32.89 | 55.68 | 129, 123, 119 |
| 58 | Tiger | Yellow | 11.72 | 61.76 | 224, 184, 35 |
| 59 | Tree | Brown | 16.78 | 70.92 | 119, 72, 32 |
| 60 | Turtle | Brown | 21.33 | 64.25 | 111, 80, 44 |
| 62 | Water | White | 22.00 | 42.84 | 250, 249, 249 |
| 63 | Watermelon | Red | 35.81 | 71.15 | 222, 51, 82 |
| 64 | Whale | Blue | 35.81 | 69.92 | 54, 76, 145 |

*Note.* Supplementary C1 presents the second most frequently associated color for words that elicited more than one color association. Numbers correspond to the numbering used in Supplementary A1 or Supplementary A2. Percentage of agreement refers to the proportion of participants who associated the word with the listed secondary color. Mean association strength reflects the average rating of the strength of the relation between the word and that secondary color. RGB values represent the average color selected by participants for that association. These values should be interpreted as secondary associations and should be considered when selecting incongruent color-word pairings.

**Supplementary C2**

***Third Most Frequently Associated Colors***

| **No.** | **Word** | **Associated Color** | **% Of Agreement** | **Mean Association Strength** | **RGB Values** |
| --- | --- | --- | --- | --- | --- |
| **Dataset words** | | | | | |
| 28 | Crocodile | Brown | 7.23 | 60.72 | 124, 111, 87 |
| 31 | Dirt | Grey | 9.45 | 66.78 | 101, 99, 98 |
| 64 | Seal | Blue | 6.20 | 75.44 | 91, 167, 223 |
| **Non-dataset words** | | | | | |
| 8 | Blueberries | Black | 6.00 | 78.00 | 20, 12, 41 |
| 9 | Bomb | Orange | 5.44 | 64.75 | 223, 118, 21 |
| 12 | Cancer | Red | 14.58 | 36.90 | 192, 29, 28 |
| 13 | Cantaloupe | Green | 21.62 | 56.37 | 91, 175, 61 |
| 14 | Cashew | White | 11.80 | 58.72 | 239, 235, 225 |
| 20 | Eagle | White | 11.40 | 62.40 | 248, 247, 247 |
| 21 | Fire | Yellow | 10.20 | 75.46 | 236, 204, 45 |
| 24 | Giraffe | Brown | 24.30 | 65.88 | 143, 94, 41 |
| 25 | Goat | Grey | 22.29 | 63.20 | 158, 152, 149 |
| 26 | Grapefruit | Orange | 15.03 | 71.60 | 232, 142, 53 |
| 27 | Grapes | Red | 6.75 | 60.70 | 176, 23, 30 |
| 28 | Hand Grenade | Black | 18.91 | 66.57 | 18, 14, 14 |
| 29 | Iron | Orange | 6.08 | 68.68 | 215, 117, 6 |
| 32 | Lava | Black | 6.71 | 78.30 | 55, 31, 23 |
| 33 | Lion | Orange | 24.13 | 67.25 | 216, 141, 39 |
| 35 | Lizard | Grey | 6.04 | 69.55 | 117, 114, 99 |
| 36 | Lobster | Orange | 11.25 | 66.58 | 230, 110, 19 |
| 37 | Mango | Green | 14.58 | 67.04 | 77, 163, 61 |
| 39 | Moon | Yellow | 11.56 | 68.11 | 234, 226, 91 |
| 40 | Mouse | White | 15.89 | 71.43 | 251, 251, 251 |
| 44 | Onion | Yellow | 9.45 | 59.64 | 236, 229, 146 |
| 45 | Peach | Yellow | 8.72 | 67.84 | 240, 197, 65 |
| 50 | Potato | Yellow | 9.52 | 68.57 | 228, 211, 107 |
| 52 | Radish | Purple | 10.13 | 71.86 | 150, 37, 128 |
| 53 | Rat | Black | 9.52 | 74.64 | 26, 21, 21 |
| 56 | Sand | White | 8.90 | 62.69 | 236, 232, 217 |
| 60 | Turtle | Grey | 14.00 | 46.09 | 91, 89, 90 |
| 63 | Watermelon | Pink | 20.27 | 68.86 | 239, 84, 137 |
| 64 | Whale | Black | 12.83 | 68.00 | 25, 22, 25 |

*Note.* Supplementary C2 presents the third most frequently associated color for words with an additional color association. Numbers correspond to the numbering used in Supplementary A1 or Supplementary A2. Percentage of agreement refers to the proportion of participants who associated the word with the listed tertiary color. Mean association strength reflects the average strength rating for that tertiary association. RGB values represent the average color selected by participants for that association. Because these are third-level associations, they are expected to be less frequent and less dominant than the primary and secondary associations. They are included mainly to support transparency and stimulus control when constructing future experiments.

**Supplementary D** Supplementary D provides a summary-level visualization of the mean reported association strength for each color category (see Figure S2). Whereas Supplementary A, Supplementary C1, and Supplementary C2 provide item-level information for individual words, Supplementary D summarizes the broader pattern of association strength across the 11 color categories. This figure allows readers to examine whether words associated with some colors were, on average, rated as more strongly color-related than words associated with other colors. For each color category, association strength was calculated by averaging the strength ratings of the words whose primary associated color belonged to that category. Thus, the figure provides an overview of how strongly each color category was represented in the stimulus pool. This information may be useful for researchers who wish to select stimuli not only at the individual-word level, but also at the level of broader color categories. However, because there is variability among words within each category, this figure should be interpreted alongside the item-level information provided in Supplementary A.

**Figure S2**
***Mean Reported Strength of Association for Each Color Category***

**Note.** Supplementary D presents the mean reported strength of association for each color category. For each word, the relevant color category was determined by the most frequently reported associated color. Bars represent the mean association strength for words within each color category, and error bars represent one standard error above and below the mean. Values displayed above the bars indicate the mean association strength and standard deviation for each color category. This figure provides a summary-level overview of the dataset and should be interpreted together with the item-level information reported in Supplementary A.

 To examine whether the strength of word-color associations differed across color categories, we conducted a one-way ANOVA with color category as a between-items factor and mean association strength as the dependent variable. Mean association strength differed significantly across color categories, F(10, 132) = 1.91, p = .049, $\eta_{p}^{2}$= .126. Descriptively, the strongest associations were observed for red words (*M* = 86.6, *SD* = 6.81), whereas the weakest associations were observed for brown words (*M* = 73.8, *SD* = 9.07). This difference was statistically significant, *t*(132) = -4.11, *p* < .001, indicating that red-associated words were rated as more strongly associated with their corresponding color than brown-associated words. This finding exemplifies the variability in association strength across color categories: some color categories were represented by relatively strong and consistent word-color associations, whereas others showed weaker average associations. This pattern reinforces the importance of reporting both item-level and category-level information, because the strength of color associations cannot be assumed to be uniform across all colors. Full descriptive statistics by color category and the post-hoc comparisons indicating which color categories differed from one another are provided in the analysis script, which is available at OSF.

**Supplementary E** Supplementary E provides the complete stimulus-list structure used in the semantic Stroop validation experiments. Each participant in Experiments 2 and 3 was randomly assigned to one of 10 stimulus lists. Each list included four response colors, with four color-associated words assigned to each response color, resulting in 16 unique word-color pairings per list. Each word appeared once in a congruent color and once in an incongruent color, producing 32 base stimuli per list: 16 congruent stimuli and 16 incongruent stimuli. The full structure of the 10 stimulus lists is presented in Figure S3. The main rationale for this list-construction procedure was to create a manual semantic Stroop task that was balanced, manageable for participants, and directly linked to the validated color-association measures from Experiment 1. Because manual responses require participants to learn a limited number of response keys, each list included only four response colors. These four colors served as the response alternatives throughout the task, ensuring that every congruent and incongruent stimulus could be classified using one of the four trained response keys.
 For each word, the congruent color was defined as the primary associated color identified in Experiment 1. The exact RGB value used to display the congruent stimulus was based on the mean RGB value selected by participants for that word in Experiment 1. Incongruent stimuli were created by presenting the same word in a color belonging to another color category from the same list. Importantly, the incongruent colors were not arbitrary generic color values; rather, they were taken from the RGB values of other validated stimuli in the same list. In addition, when constructing the incongruent pairings, we ensured that a word was never paired with a color that participants in Experiment 1 had ranked as meaningfully associated with that object. Thus, the incongruent colors were selected to be unrelated to the target word based on the association responses from Experiment 1, while still being valid response colors within the same list.
 At the trial level, the 32 base stimuli in each list were repeated across the experiment to create 256 experimental trials per participant. Thus, each word appeared 32 times in total: 16 times in its congruent color and 16 times in its incongruent color. This repetition was necessary to obtain stable reaction-time estimates for each word and each congruency condition while keeping the task length reasonable for online testing.
 Some words also appear in more than one list. This cross-list repetition was necessary because the number of available words was not identical across color categories. Some color categories included many eligible words, whereas others included fewer words that could be used after excluding black-associated words and applying the constraints of the four-color list structure. Because each list required four colors and four words per color, it was not possible to construct all 10 lists with completely unique words while maintaining balanced response alternatives within each list. Therefore, a small number of stimuli were repeated across lists to preserve the balance of four response colors, equal numbers of words per color within each list, and equal numbers of congruent and incongruent trials. These repeated stimuli were not repeated within the same list as separate items; rather, they were used in more than one list to satisfy the broader list-construction constraints.

**Figure S3
 *Stimulus-List Structure Used in Experiments 2 and 3*** **Note.** Each panel presents one of the 10 stimulus lists used in Experiments 2 and 3. Each list included 16 unique words, with each word appearing once in a congruent color and once in an incongruent color. The congruent pairing reflects the word’s primary associated color from Experiment 1, whereas the incongruent pairing reflects the unrelated color assigned to that word within the same list.

The full Supplementary E sheet provides the trial-level structure for each of the 10 lists. After removing black-associated words, 136 words from Experiment 1 were eligible for the validation experiments (76 dataset words and 60 non-dataset words). One additional word, Lilach,^^[[1]](#footnote-1)^^ was added only for list-construction purposes, bringing the full list-construction pool to 137 words. Black-associated words were removed because the semantic Stroop task was presented on a black background, which made black unsuitable as a response color and as a stimulus color in the manual color-identification task. For each list, the Supplementary E sheet includes the word, the displayed color, RGB values, hex value, congruency condition, association-strength value, list number, correct response key, and instruction image used for that list. This information is included to make the validation experiments fully reproducible and to allow researchers to reuse the exact stimulus structure.

## **Additional Study Material Files Available on OSF**

In addition to the supplementary tables reported in this document, two additional files are provided on OSF together with the study materials, data, and analysis code (see the following link: <https://osf.io/twqys/?view_only=5a1b08637fd743838dfa5ce8070f0e29>). The first file**, Full Item-level Data.xlsx,** contains the full item-level output from Experiment 1. This file includes the first, second, and third most frequently reported color associations for each word, the percentage of participants selecting each associated color, the corresponding RGB values, the number of participants contributing to each color estimate, and the mean and standard deviation of the association-strength ratings. It also includes the broader response summaries used to construct the tables reported in Supplementary A and Supplementary C. The second file, **Summary of RGB values.xlsx**, provides a summary of the RGB values reported by participants for each word-color association. Specifically, it summarizes the red, green, and blue values selected by participants for each word and color category, along with the number of observations contributing to each estimate. This file is intended to allow researchers to inspect the empirical color values underlying the representative RGB values reported in the Supplementary Materials and to reuse or adapt these values for future stimulus construction.

1. The word Lilach (see List 3) was not included in Experiment 1 and was added only for list-construction purposes. After removing black-associated words, 136 words from Experiment 1 were eligible for the validation experiments: 76 dataset words and 60 non-dataset words. Lilach was added as one additional stimulus because the number of available purple-associated words was limited to three, whereas each list required four words for each response color. Thus, Lilach was included to complete the set of four purple-associated words in the relevant list and to preserve the balanced four-color list structure. It was not included in the item-level validation analyses. [↑](#footnote-ref-1)
